# Supplementary material for: Spiritual leadership and service performance among Chinese flight attendants: The mediating effects of meaningful work and work engagement
Source: PLoS One. 2026 May 21;21(5):e0324405. doi: 10.1371/journal.pone.0324405 (PMC13193339; doi:10.1371/journal.pone.0324405)
Supplement: Attachment — Submitted filename: PONE-D-25-18162_reviewer.pdf [file pone.0324405.s002.pdf]

PLOS ONE

Spiritual leadership and service performance among Chinese flight attendants: The mediating effects of meaningful work and work engagement

--Manuscript Draft--

**Manuscript Number:**	PONE-D-25-18162
**Article Type:**	Research Article
**Full Title:**	Spiritual leadership and service performance among Chinese flight attendants: The mediating effects of meaningful work and work engagement
**Short Title:**	Spiritual leadership and service performance among Chinese flight attendants: mediating effects
**Corresponding Author:**	Xuqun You<br>Shanxi Normal University<br>Xi'an City, Shaanxi Province, CHINA
**Keywords:**	Key words: Flight attendants, Spiritual leadership, Meaningful work, Work engagement, Service performance
**Abstract:**	As an irreplaceable part of flight crew for passenger carriage in big aircraft, flight attendants are responsible for cabin safety, security, passenger service and emergency during the flight. However, as the main interface between passengers and airlines, flight attendants' delivering of high quality service is crucial in retention of passengers' loyalty and airlines' prosperity. Hence, how to promote flight attendants' service performance during the flight is important in airline management. The main purpose of this study was to explore the roles of spiritual leadership, meaningful work, work engagement and flight attendants' service performance in the sample of 313 Chinese flight attendants from Tibet Airlines. and its branches. Correlation analysis indicated that spiritual leadership, meaningful work, work engagement and service performance were significantly correlated with each other. Bootstrap analysis showed that meaningful work and work engagement not merely play a simple mediating role between spiritual leadership and service performance, but jointly play a chain mediating role. Theoretical implications for this research and managerial suggestions for how to improve flight attendants' service performance in airline management are discussed.
**Order of Authors:**	Lei DU
	Ying Li
	Zixian Li
	Tao Wen
	Fei Gu
	Yan Pan
	Ming Ji
	Saifang Liu
	Jun Zhang
	Mingliang Li
	Yuan Li
	Yaoliang Wu
	Chenguang Pan
	Xuqun You
**Additional Information:**	
**Question**	**Response**

Powered by Editorial Manager® and ProduXion Manager® from Aries Systems Corporation

**Financial Disclosure**<br><br>Enter a financial disclosure statement that describes the sources of funding for the work included in this submission. Review the submission guidelines for detailed requirements. View published research articles from *PLOS ONE* for specific examples.<br><br>This statement is required for submission and **will appear in the published article** if the submission is accepted. Please make sure it is accurate.<br><br>**Funded studies**<br>Enter a statement with the following details:<br>• Initials of the authors who received each award<br>• Grant numbers awarded to each author<br>• The full name of each funder<br>• URL of each funder website<br>• Did the sponsors or funders play any role in the study design, data collection and analysis, decision to publish, or preparation of the manuscript?<br><br>Did you receive funding for this work?	Yes
Please add funding details.<br>as follow-up to "**Financial Disclosure**<br><br>Enter a financial disclosure statement that describes the sources of funding for the work included in this submission. Review the submission guidelines for detailed requirements. View published research articles from *PLOS ONE* for specific examples.<br><br>This statement is required for submission and **will appear in the published article** if the submission is accepted. Please make sure it is accurate.	National Natural Science Foundation of China (32000753)<br>Key Research and Development Program of Shaanxi (2021SF-481)<br>Young Talent Fund of University Association for Science and Technology in Shaanxi (202008)

Powered by Editorial Manager® and ProduXion Manager® from Aries Systems Corporation

**Funded studies**<br>Enter a statement with the following details:<br>• Initials of the authors who received each award<br>• Grant numbers awarded to each author<br>• The full name of each funder<br>• URL of each funder website<br>• Did the sponsors or funders play any role in the study design, data collection and analysis, decision to publish, or preparation of the manuscript?<br><br>Did you receive funding for this work?"	
Please select the country of your main research funder (please select carefully as in some cases this is used in fee calculation).<br>as follow-up to "**Financial Disclosure**<br><br>Enter a financial disclosure statement that describes the sources of funding for the work included in this submission. Review the submission guidelines for detailed requirements. View published research articles from *PLOS ONE* for specific examples.<br><br>This statement is required for submission and **will appear in the published article** if the submission is accepted. Please make sure it is accurate.<br><br>**Funded studies**<br>Enter a statement with the following details:<br>• Initials of the authors who received each award<br>• Grant numbers awarded to each author<br>• The full name of each funder<br>• URL of each funder website<br>• Did the sponsors or funders play any role in the study design, data collection and analysis, decision to publish, or preparation of the manuscript?<br><br>Did you receive funding for this work?"	CHINA - CN
**Competing Interests**	The authors declared that no competing interests exist.

Powered by Editorial Manager® and ProduXion Manager® from Aries Systems Corporation

Use the instructions below to enter a competing interest statement for this submission. On behalf of all authors, disclose any competing interests that could be perceived to bias this work—acknowledging all financial support and any other relevant financial or non-financial competing interests.<br><br>This statement is required for submission and **will appear in the published article** if the submission is accepted. Please make sure it is accurate and that any funding sources listed in your Funding Information later in the submission form are also declared in your Financial Disclosure statement.<br><br>View published research articles from *PLOS ONE* for specific examples.<br><br>**NO authors have competing interests**<br><br>Enter: *The authors have declared that no competing interests exist.*<br><br>**Authors with competing interests**<br><br>Enter competing interest details beginning with this statement:<br><br>*I have read the journal's policy and the authors of this manuscript have the following competing interests: [insert competing interests here]*<br><br>* typeset	
**Ethics Statement**<br><br>Enter an ethics statement for this submission. This statement is required if the study involved:<br><br>• Human participants<br>• Human specimens or tissue<br>• Vertebrate animals or cephalopods<br>• Vertebrate embryos or tissues	This study was conducted in accordance with the Declaration of Helsinki and was approved by the Human Research Ethics Committee of Shaanxi Normal University, and informed consent was obtained from all individual participants included in this study.

• Field research<br><br>Write "N/A" if the submission does not require an ethics statement.<br><br>General guidance is provided below. Consult the submission guidelines for detailed instructions. **Make sure that all information entered here is included in the Methods section of the manuscript.**	

Powered by Editorial Manager® and ProduXion Manager® from Aries Systems Corporation

**Format for specific study types**<br><br>**Human Subject Research (involving human participants and/or tissue)**<br>• Give the name of the institutional review board or ethics committee that approved the study<br>• Include the approval number and/or a statement indicating approval of this research<br>• Indicate the form of consent obtained (written/oral) or the reason that consent was not obtained (e.g. the data were analyzed anonymously)<br><br>**Animal Research (involving vertebrate animals, embryos or tissues)**<br>• Provide the name of the Institutional Animal Care and Use Committee (IACUC) or other relevant ethics board that reviewed the study protocol, and indicate whether they approved this research or granted a formal waiver of ethical approval<br>• Include an approval number if one was obtained<br>• If the study involved *non-human primates*, add *additional details* about animal welfare and steps taken to ameliorate suffering<br>• If anesthesia, euthanasia, or any kind of animal sacrifice is part of the study, include briefly which substances and/or methods were applied<br><br>**Field Research**<br><br>Include the following details if this study involves the collection of plant, animal, or other materials from a natural setting:<br>• Field permit number<br>• Name of the institution or relevant body that granted permission	
**Data Availability**<br><br>Authors are required to make all data underlying the findings described fully available, without restriction, and from the time of publication. PLOS allows rare exceptions to address legal and ethical concerns. See the PLOS Data Policy and FAQ for detailed information.	No - some restrictions will apply

Powered by Editorial Manager® and ProduXion Manager® from Aries Systems Corporation

A Data Availability Statement describing where the data can be found is required at submission. Your answers to this question constitute the Data Availability Statement and **will be published in the article**, if accepted.<br><br>**Important:** Stating 'data available on request from the author' is not sufficient. If your data are only available upon request, select 'No' for the first question and explain your exceptional situation in the text box.<br><br>Do the authors confirm that all data underlying the findings described in their manuscript are fully available without restriction?	
**Describe where the data may be found in full sentences. If you are copying our sample text, replace any instances of XXX with the appropriate details.**<br><br>• If the data are **held or will be held in a public repository**, include URLs, accession numbers or DOIs. If this information will only be available after acceptance, indicate this by ticking the box below. For example: *All XXX files are available from the XXX database (accession number(s) XXX, XXX.).*<br>• If the data are all contained **within the manuscript and/or Supporting Information files**, enter the following: *All relevant data are within the manuscript and its Supporting Information files.*<br>• If neither of these applies but you are able to provide **details of access elsewhere**, with or without limitations, please do so. For example:<br><br>*Data cannot be shared publicly because of [XXX]. Data are available from the XXX Institutional Data Access / Ethics Committee (contact via XXX) for researchers who meet the criteria for access to confidential data.*<br><br>*The data underlying the results presented in the study are available from (include the name of the third party*	All relevant data are within the manuscript and its Supporting Information files.

and contact information or URL).<br>• This text is appropriate if the data are owned by a third party and authors do not have permission to share the data.<br><br>* typeset	
Additional data availability information:	Tick here if the URLs/accession numbers/DOIs will be available only after acceptance of the manuscript for publication so that we can ensure their inclusion before publication.; Tick here if your circumstances are not covered by the questions above and you need the journal's help to make your data available.

Manuscript

Click here to access/download;Manuscript;Manuscript2025.docx

Spiritual leadership and service performance among Chinese flight attendants: The mediating effects of meaningful work and work engagement

Lei Du [a, b, c], Ying Li [a, b], Zixian Li [d], Tao Wen [c], Fei Gu [c], Yan Pan [a, b], Ming Ji [a, b], Saifang Liu [a, b], Jun Zhang [a, b e], Mingliang Li [a,b], Yuan Li [a, b], Yaoliang Wu [a, b, f], Chenguang Pan [g], Xuqun You [a, b, *]

Order of Authors:

1. Lei Du, Ph.D. candidate, [a] School of Psychology, Shaanxi Normal University, Xi'an, Shaanxi 710062, China; [b] Shaanxi Provincial Key Laboratory of Behavior and Cognitive Neuroscience, Xi'an, Shaanxi 710062, China; [c] Flight Department, Tibet Airlines Co., Ltd., Chengdu Branch, Chengdu, Sichuan 610200, China; E-mail address: duleilanl@163.com

2. Ying Li, Ph.D. candidate, [a] School of Psychology, Shaanxi Normal University, Xi'an, Shaanxi 710062, China; [b] Shaanxi Provincial Key Laboratory of Behavior and Cognitive Neuroscience, Xi'an, Shaanxi 710062, China; E-mail address: 920675143@qq.com

3. Zixian Li, Flight attendant, [d] Cabin Service Department, Tibet Airlines Co., Ltd., Chengdu Branch, Chengdu, Sichuan 610200, China; E-mail address: 1197657806@qq.com

4. Tao Wen, Vice president, [c] Flight Department, Tibet Airlines Co., Ltd., Chengdu Branch, Chengdu, Sichuan 610200, China; E-mail address: 564975938@qq.com

5. Fei Gu, Manager, [c] Flight Department, Tibet Airlines Co., Ltd., Chengdu Branch, Chengdu, Sichuan 610200, China; E-mail address: 418719883@qq.com

6. Yan Pan, M.D. candidate, [a] School of Psychology, Shaanxi Normal University, Xi'an, Shaanxi 710062, China; [b] Shaanxi Provincial Key Laboratory of Behavior and Cognitive Neuroscience, Xi'an, Shaanxi 710062, China; E-mail address: pany2018@126.com

7. Ming Ji, Ph.D., [a] School of Psychology, Shaanxi Normal University, Xi'an, Shaanxi 710062, China; [b] Shaanxi Provincial Key Laboratory of Behavior and Cognitive Neuroscience, Xi'an, Shaanxi 710062, China; E-mail address: jiming@snnu.edu.cn

8. Saifang Liu, Ph.D. candidate, [a] School of Psychology, Shaanxi Normal University, Xi'an, Shaanxi 710062, China; [b] Shaanxi Provincial Key Laboratory of Behavior and Cognitive Neuroscience, Xi'an, Shaanxi 710062, China; E-mail address:1915479947@qq.com

9. Jun Zhang, Ph.D candidate, [a] School of Psychology, Shaanxi Normal University, Xi'an, Shaanxi 710062, China; [b] Shaanxi Provincial Key Laboratory of Behavior and Cognitive Neuroscience, Xi'an, Shaanxi 710062, China; [e] Flight Standard Department, Central and Southern Regional Administration of CAAC, No.163,Yunxiao Road,Baiyun District, Guangzhou, Guangdong Province, China; E-Address: zj_csn@163.com

10. Mingliang Li, Ph.D candidate, [a] School of Psychology, Shaanxi Normal University, Xi'an, Shaanxi 710062, China; [b] Shaanxi Provincial Key Laboratory of Behavior and Cognitive Neuroscience, Xi'an, Shaanxi 710062, China E-mail address: limingliang_xn@caac.gov.cn

11. Yuan Li, Ph.D., [a] School of Psychology, Shaanxi Normal University, Xi'an, Shaanxi 710062, China; [b] Shaanxi Provincial Key Laboratory of Behavior and Cognitive Neuroscience, Xi'an, Shaanxi 710062, China; E-mail address: psyliyuan@163.com

12. Yaoliang Wu, Ph.D. candidate, [a] School of Psychology, Shaanxi Normal University, Xi'an, Shaanxi 710062, China; [b] Shaanxi Provincial Key Laboratory of Behavior and Cognitive Neuroscience, Xi'an, Shaanxi 710062, China; [f] Flight Department, China Eastern Airlines Co., Ltd., Anhui Branch, Hefei, Anhui 230031, China; E-mail address: 942896478@qq.com

13. Chenguang Pan, Captain, [g] Flight Department, China Eastern Airlines Co..Ltd., Shanghai 201100, China; E-mail address: 196434996@qq.com

14. Xuqun You, Ph.D., [a] School of Psychology, Shaanxi Normal University, Xi'an, Shaanxi 710062, China; [b] Shaanxi Provincial Key Laboratory of Behavior and Cognitive Neuroscience, Xi'an, Shaanxi 710062, China; E-mail address: xunqunyoucaac@163.com

Corresponding author:

*1. Xuqun You. (E-mail address: xunqunyoucaac@163.com; Full postal address: 199 Chang'an South Road, Xi'an City, Shaanxi Province, 710062)

Abstract: As an irreplaceable part of flight crew for passenger carriage in big aircraft, flight attendants are responsible for cabin safety, security, passenger service and emergency during the flight. However, as the main interface between passengers and airlines, flight attendants' delivering of high quality service is crucial in retention of passengers' loyalty and airlines' prosperity. Hence, how to promote flight attendants' service performance during the flight is important in airline management. The main purpose of this study was to explore the roles of spiritual leadership, meaningful work, work engagement and flight attendants' service performance in the sample of 313 Chinese flight attendants from Tibet Airlines. and its branches. Correlation analysis indicated that spiritual leadership, meaningful work, work engagement and service performance were significantly correlated with each other. Bootstrap analysis showed that meaningful work and work engagement not merely play a simple mediating role between spiritual leadership and service performance, but jointly play a chain mediating role. Theoretical implications for this research and managerial suggestions for how to improve flight attendants' service performance in airline management are discussed.

Key words: Flight attendants, Spiritual leadership, Meaningful work, Work engagement, Service performance

1. Background

Flight attendants are the essential and important part of the flight crew for the flight operation of carriage of passengers in large aircraft, and are responsible for cabin safety, security, passenger service and emergency during the flight [1]. Currently, flying is a common method of traveling, especially in long distance trip, rather than a luxurious form of travel, like in the mid-twentieth century [2]. According to the International Air Transport Association (IATA)'s data, in 2019, the world's airlines provided beyond 4.5 billion passengers and the freedom to travel over a global network of more than 22,000 unique city pairs on 46.8 million flights. Tourists traveling internationally by air estimated to have spent about 900 billion US dollars [3]. Though civil aviation industry has suffered devastated and unprecedented losses because of worldwide COVID-19 pandemic, it is still resilient and on the rise. After the worst downturn, civil aviation industry has turned the corner on the COVID-19 pandemic [4]. Thus, How to survive and succeed in today's intense competitive air transport market and be ready for welcoming pre-pandemic levels of flight demand deserves deep study. Recent literature demonstrated that delivering of high quality service is crucial and a powerful instrument to achieve that, because in this way their passengers' satisfaction and loyalty will be enhanced [5-9]. As the main interface between passengers and airline companies, cabin attendants are the front-line service employee and critical role reflecting the image of consistent brand promise [10, 11], even when facing circadian dysrhythmia, role overload, emotional dissonance, burnout, impoliteness and dysfunctional passengers [1, 7, 12-15]. Considering their job is full of complexities and diversities [16], therefore, how to effectively and efficiently promote cabin attendants' service performance during the flight is an intractable challenge in airline management. Yet limited empirical studies could be referred to. This article enriches the occupational psychological exploration about service performance.

Current limited research on service performance mainly focus on individual perspective, like personality and motivation [16, 17], self-efficacy [18], psychological capital [19, 20], job tenure and burnout [12]. However, little research has been conducted in the perspective of organizational management, even if service performance is the key for airlines to realize their business strategic goals [21]. As an effective tool to organize collective effort, leadership is important for organizational effectiveness, thriving and prosperity, as well as financial and psychological well-being of the incumbents [22]. Among different leadership theories, the reason why spiritual leadership has been considered in this study and be deemed to positively affect service performance is because that it creates vision, attitudes and value congruence across the the organization and individual levels to develop higher levels of intrinsic motivation, organizational commitment and work performance [23],which differentiates spiritual leadership from other positive leadership styles, e.g.,servant leadership [24], ethical leadership [25], transformational leadership and transactional leadership [26]. Spiritual leadership is receiving more attention recently and has been proved to work well in promoting task performance and proactive work behaviors for fuel workers [27] and safety performance for airline pilots [28]. However, to the best of our knowledge, there is no empirical study that has examined the relationship between the spiritual leadership and service performance in the sample of flight attendants to the date. Therefore, this article gauges the association between them and contributes to the existing knowledge of leadership theory.

Nowadays, meaningful work is about a positive psychology state whereby employees have a feeling of making a positive, important and meaningful contribution through their work for a worthwhile purpose [29]. According to Hackman and Oldham's Job Characteristics Theory (JCT) [30], meaningful work is a critical psychological factor leading to quality job performance, higher job satisfaction and lower withdrawal intentions [31-33]. Thus, understanding how to create, manage, maintain and enhance meaningful work would provide

powerful capability for achieving optimum and sustainable work performance for individuals (i.e., better service performance of cabin attendants during the flight) and organizations (i.e., succeed in competitive aviation market) [34]. Meanwhile, spiritual leadership simultaneously utilizes spiritual, social, and, ethical values and rational determinants to create a working environment and culture characterized by autonomous intrinsic motivation, positive mental state, voluntarily helping others and participating decision making [23, 35, 36], through which individual's job performance is enhanced [28, 37, 38]. Inspired by potential positive psychological relationship between spiritual leadership, meaningful work and service performance, the meaningful work was chosen as a mediator between the spiritual leadership and service performance. However, the current literature lacks empirical studies relating the psychological mechanism between the spiritual leadership and service performance [36]. Therefore, this study firstly further extend the research on interrelations between spiritual leadership and service performance in order to uncover how spiritual leadership affects service performance.

As a proximal outcome of meaningful work, work engagement - a positive, fulfilling, job related psychological state that is characterized by vigor, dedication and absorption [39] - has much deeper relations with meaningful work than others, such as mental and physical health, work performance, withdrawal intentions and organizational citizenship behaviors [32, 40]. Furthermore, some scholars even view work engagement as components of meaningful work [41], because the mental state of employees engaging in meaningful work melds with the cognitive and motivational components of work engagement [39, 42-44]. Based on Job Demands–Resources (JD–R) theory [45], spiritual leadership has potential to simulate job resource (e.g. supervisor support and colleague support) and personal resource (e.g., optimism, hope), furtherly lead to the satisfaction of psychological needs (e.g., meaningfulness) that in turn lead to work engagement and job performance in the end [32]. However, current

preponderance of empirical studies emphasized that meaningful work could positively predict work engagement [42, 44, 46-48] and work engagement could positively predict in-role and extra-role performance of flight attendants [8, 12, 20] and flight attendants' service performance [19, 49], but no empirical research has examined the mediator role of work engagement between spiritual leadership and service performance, let alone meaningful work and work engagement acting as mediator variables together.

In general, thus far, this study is the first to investigate previously mentioned relationships simultaneously in the perspective of organizational psychology, positive psychology, and occupational psychology using data gathered from airlines' flight attendants. The theoretical chain mediation model of this study is depicted in Fig. 1. By doing so, this study not only advances the literature on the relationship between spiritual leadership and service performance, but also foregrounds and extends the psychological mechanism through which spiritual leadership weaves its favorable impact on flight attendants' service performance so that airlines' managers and leaders could have clues in ameliorating management.

Fig. 1. The proposed chain mediation model.

2. Literature review and hypotheses

2.1 Spiritual leadership and service performance

In the economic globalization, today's intensive competitive civil aviation industry has evolved from its traditional role as a transportation method into a service industry [50]. Hence, delivery of high quality service and retention of loyal passengers are the crucial factors in surviving and being succeed in such a large and prosperous market [6]. High -quality service during the flight could provide a great advantage for airlines in distinguishing it from competitors and harvesting customer loyalty [51]. However, Flight attendants act a vital role in

that process [7, 10, 15, 52], because they are the ones having more contact with passengers than any other employees of airlines [50], and not only do they impress people by their elegant professionalism, gracious service [50], but also convey the brand image and promise of airlines [11, 49, 52], even when beset with internal difficulties and external difficulties, such as physical and mental stress, emotional labor, burnout, fatigue, irregular work schedule, long night shifts, disruptive passengers [14, 15, 50, 53-55]. Thus, flight attendants' work is complex and diversified, those who are unable to realize their duties during the flight would cause service failure and customer defections [56]. Under these intricate scenarios, finding a way to establish and maintain a type of work environment in which flight attendants are motivated to better realize their job duties would significantly contribute to an effective airline management [7].

One of the most complex and discussed social processes in anywhere is leadership [57]. Leadership is not only about power and authority but also about emotional connections and spiritual values [58], which is the art of management. There are growing calls to study different leaderships, e.g., transformational leadership [59, 60], empowering leadership [37] and safety leadership [61], in association with job performance (e.g., safety performance for pilots) to deep and better comprehend the effectiveness of leadership for solving employees' performance issues [28]. This constitutes a serious omission that, as an indispensable part of flight crew, flight attendants' job performance (e.g., service performance), and especially its link with leadership, have received little attention. The present investigation fills this research void by studying the links between spiritual leadership and service performance.

Spiritual leadership theory was built upon Giacalone and Jurkiewicz's [62] definition of workplace spirituality, and was extended by Fry [23]. Spiritual leadership is defined as the values, attitudes, and behaviors that are necessary to intrinsically motivate one's self and others, incorporating with vision, hope/faith, altruistic love, theories of workplace spirituality and sense of spiritual survival/well-being, through calling and membership [23]. The reason to

choose spiritual leadership as antecedent variable is because spiritual leadership involves motivating and inspiring workers through a transcendent vision and a corporate culture, and satisfying the fundamental needs of both leader and followers for spiritual well-being [35]; and, ultimately, fostering higher level of employees' well-being, organizational commitment, productivity and excellent job performance [27, 62-65], which meets the needs of airline management. Furthermore, spiritual leaders implant employees with trust, intrinsic motivation, and commitment that is necessary to simultaneously optimize organizational performance, meanwhile, excellent organizational outputs, e.g., high service quality and on-time delivery, would affect customer satisfaction which, in turn, ultimately affect financial performance of airlines [66]. Crossman [67] believes that spiritual leadership has the potential to emerge as a powerful and courageous innovative management paradigm for better human well-being and performance excellence in the twenty-first century organizations. From empirical evidence point of view, spiritual leadership has been proved to work well in promoting pilots' safety performance, no matter in direct effects or indirect effects [28]. Same significantly positive effects of spiritual leadership have also been found by Chen and Yang [63] in facilitating employees to perform excellent organizational citizenship behaviors in finance and retail service industries. Moreover, in energy industry, Wang et al. [27] found that the spiritual leadership is positively related with employees' task performance, knowledge sharing behaviors and innovation behavior. Additionally, Yang et al. [36] found that when perceiving a sense of organizational membership from spiritual leadership provided by their leaders, employees are more likely to reciprocate by improving their job performance, which is also mirrored in the empirical study of hotel industry by Salehzadeh et al. [68]. Therefore, the following hypothesis were proposed:

Hypothesis 1. Spiritual leadership is positively related with flight attendants' service performance

2.2 Mediating role of Meaningful work

Limited studies over the world suggest that spiritual leadership plays a pivotal role in promoting employees' job performance and organizational performance, yet there is little understanding of how spiritual leadership enhances employees' job performance, or more specifically flight attendants' service performance, which refers to interpersonal behaviors that contribute to favorable work outcomes by serving customers [17, 69-71]. Different with other leadership theories, spiritual leadership consists of three fundamental components that are vision, hope/faith, and altruistic love, as the leader's values, attitudes, and behaviors, separately [27]. Vision refers to a meaningful future, determining the organizational goals, reflecting organizational ambitions, giving meaning to work and encouraging hope and faith [68, 72, 73]. Altruistic love brings high organization commitment and productivity, meanwhile hope/faith gives employees a belief that their vision will be achieved, and thus being engaged [23, 74, 75]. Furthermore, all of which are manifested through calling and membership, and calling is also about meaning [76]. As a result, employees' intrinsic motivation for pursuit of better job performance is boosted by helping employees feel that the work is meaningful [36, 63].

People not only commit to pursuing competence in their work, but also social value and work meaningfulness [77]. Scholars have defined meaningful work in multiple ways and there are some different interchangeable terms, such as meaningful work, meaningfulness and meaning in work, which are all referring to the significance and value of work with positive valence [78]. Drawing upon job characteristics theory [30, 79], five job dimensions, i.e. , skill variety, work identity, work significance, autonomy, and feedback, generate three critical psychological states, and one of which is meaningful work. Meaningful work is an internal work motivation propelling employees toward goal-orientated behaviors and further resulting in higher job satisfaction, quality job performance, lower absenteeism and other beneficial work outcomes [32, 80]. Kahn [42] extended theory of job characteristics, and viewed meaningful

work as employees' consciousness to encompass deep aspects of themselves, such as expression of self-value and aspiration. In other words, when employees' job characteristics and tasks match their own values and individual identities, they would perceive higher level of meaningful work and thus have better work-related outcomes, such as effective management, better organizational performance, greater organizational commitment. [32, 34, 48, 81]. Further empirical evidence demonstrated that when employees perceive more meaningfulness of work made by their leaders, rather than perceiving work as an exchange process based on extrinsic rewards, they are more likely to improve their job performance in return [36, 58, 82]. On the other hand, a leader who draws on work meaningfulness is more capable to distinguish where an organization needs to go and how to get there [34]. Previous researches have found that expression of a clear vision and ways to implement that vision, through, for example transformational leadership and charismatic leadership, is positively related to employees' performance based on job characteristics theory [83, 84], and meaningful work is that vision. Furthermore, Duchon and Plowman [76] viewed meaningful work as the part of work unit spirituality, hereby offering a set of propositions for future examination about the relationships between spiritual leadership, work unit spirituality and work unit performance. However, up to date, the current literature lacks empirical studies regarding meaningful work as a mediator between spiritual leadership and flight attendants' service performance. Informed by this void in existing knowledge, considering the critical components of spiritual leadership and based on job characteristics theory, this study firstly proposes the followings:

Hypothesis 2: Spiritual leadership can positively predict meaningful work.

Hypothesis 3: Meaningful work can positively predict service performance.

Hypothesis 4: Meaningful work mediates the relationship between spiritual leadership and service performance.

2.3 Mediating role of Work engagement

Over the past two to three decades, researches on work engagement has increased rapidly, given its relationship with employees' well-being and performance [85-88]. Although a wealth of knowledge about work engagement has been accumulated, e.g.,nature, causes, and consequences of engagement, the necessity of continuous study on work engagement is still existing because of the changing world of work [89, 90]. Kahn [42] firstly brought up the concept of work engagement, in which engaged employees are described as being fully physically, cognitively, and emotionally linked with their work roles. Whereafter, the most frequently cited definition of work engagement was proposed by Schaufeli et al. [39], which was defined as a positive, fulfilling, work-related state of mind that is characterized by vigor, dedication, and absorption. Vigor means high levels of energy and mental resilience during the work, and dedication stands for being strongly involved in one's work and experiencing a feeling of significance, enthusiasm, and challenge, whereas absorption refers to being fully concentrated and happily immersed in the work roles. In short, work engagement integrates high work pleasure and motivation (dedication) with cognition, affection and high attraction (vigor, absorption), and appertains to a longer performance episode, thus, it is an excellent predictor of employees' performance [85, 91]. Although current empirical researches did sufficient work in supporting that work engagement has positively association with work outcomes, such as pursers' service recovery performance [20], flight attendants' job performance [12], service performance [49] and service behavior [19], empirical researches pertaining to the factors influencing work engagement in current knowledge is still sparse. Particularly, among limited researches on the factors influencing work engagement associated with job performance, most are done in the individual perspective, e.g., psychological capital [19, 92], job crafting [8, 93], psychological contract [49], few are from perspective of organizational management, thus lacks practicality. However, translating accumulated knowledge about engagement into practical applications, e.g., promoting individual, team and

organizational performance, is vitally significant [94, 95]. Meanwhile, Bakker and Albrecht [89] believe future research on employee engagement could usefully be devoted to fully uncovering the factors affecting engagement in specific demographic groups (e.g., millennials; older workers; people with disabilities), over specific industry sectors (public, private, non-for-profit)and in different occupations (nurses, teachers, flight attendants). Furthermore, the role of leadership in promoting work engagement has received limited empirical research attention [96], though leadership theories are diversified. For example, to our knowledge, Karatepe and Talebzadeh [20] are among the few scholars who have examined that servant leadership has been proved positively related to work engagement among airline pursers, and the same applies to transformational leadership in hospitals and industrial consultancy agencies [97-99]. This study not only refined and extended current knowledge of work engagement by introducing the relationship between spiritual leadership and service performance in the sample of flight attendants, but also discussed in the perspective of organizational psychology and airline management practice, which meets the research trends and needs of work engagement. Taken together, we hypothesize that:

Hypothesis 5: Work engagement is positively related with flight attendants' service performance.

According to the Job Demands-Resources (JD-R) theory [100], job resources are related to those physical, social, or organizational aspects of the job that may mitigate job demands which require sustained physical and mental effort, such as social support, autonomy, and skill variety that may be functional in realizing working goals and stimulating individual growth and development [8, 101-103]. The JD-R theory demonstrates that job resources would become salient and gain motivational potential when employees confront with high job demands [104], and could further affect work engagement directly and indirectly through personal resources [29, 45]. Based on theoretical framework of JD-R theory, a resourceful and visionary working

environment aroused by spiritual leadership would promote flight attendants' vision, hope/faith and altruistic love, thus they would be work-engaged at elevated levels in return. In addition to job resources, studies have shown that personal resources, such as a positive psychological state characterized by confidence to succeed, optimism about future, perseverance toward goals, sustainability, resilience and organizational-based self-esteem, are important predictors of work engagement [91, 105, 106]. Following spiritual leaders of airlines, employees would obtain a sense of care, concern, understanding, support and appreciation for self, colleagues, and supervisors, and generate a strong optimistic faith that organization's vision/goal/mission will be realized and what they are working for is full of meaningfulness. As a result, employees' intrinsic motivation to work, loyalty to the organization, sustainability to the career and organizational-based self-esteem are all well enhanced [23, 29]. In brief, via spiritual leadership, employees' job resources and personal resources are both promoted that in turn lead to positive mood and to work engagement [45, 100, 104] based on JD-R theory. Hence, such engaged employees are full of energy and self-efficacy [48, 106, 107] and in turn are competent for performing desirable downstream work outcomes, such as successfully dealing with challenging problems and tough service encounters for flight attendants [45, 106, 108]. Therefore, in view of theoretical and empirical evidence, the following hypothesis are proposed:

Hypothesis 6: Spiritual leadership is positively related with flight attendants' work engagement.

Hypothesis 7: Work engagement mediates the relationship between spiritual leadership and service performance.

2.4 Spiritual leadership, meaningful work, work engagement and service performance

As mentioned above, the JD-R theories believe job resources and personal resources would lead to the satisfaction of psychological needs (e.g., need for meaningfulness, belongingness, competence) that in turn result in positive mental state and work engagement [45, 100, 104]. A empirical research in the sample of Chinese telecommunication workers executed by Albrecht

and Su [109] found that performance feedback (as a job resource) was related with work engagement through fulfilling employees' need for meaningful work. As Steger and Dik [44] say, People prosper when engaging in meaningful work, whereas organizations prosper when their employees are similarly engaged. Among existing literature, a number of studies have focused on the consequences of meaningful work both theoretically and empirically. Kahn [42] initially argued that work meaningfulness is a necessary prerequisite to the work engagement, whose qualitative findings were confirmed in the subsequent empirical research by May et al. [47] and Ghadi et al. [46]. Furthermore, Stairs and Galpin [110] depicted clear logic and conceptual link to clarify that people who feel personally meaningful in jobs are more engaged than those who do not. From perspective of proximal outcome of meaningful work, some variables have much larger relations with meaningful work than others, such as work engagement, job satisfaction and commitment [32, 41, 44, 111]. Further, Allan et al. [32] pointed out that meaningful work relating to job performance, organizational citizenship behaviors and turnover intention through work engagement, commitment and job satisfaction, which is corroborated in the empirical research by Van Wingerden and Van der Stoep [112] on a sample of 459 professionals working at a global health technology organization. In addition, Van Wingerden and Poell [48] proposed a chain mediating model utilizing data collected from a group of Dutch teachers and revealed that meaningful work is positively linked to resilience via teachers' work engagement and job crafting sequentially. Johnson and Jiang [113] collected data from 194 respondents and discovered that a sense of meaningfulness in employees would facilitate work engagement and in turn enrich employees' lives beyond the workplace. In line with these findings we hypothesize:

Hypothesis 8: Meaningful work is positively related to flight attendants' work engagement.

In fact, an individual's real life tends to be faced with intricate and protean working environments that contain more than one factor deciding an individual's behaviors [114], which

is even more true when put into flight attendants' working environment. Although JD-R theory delineates guidance to propose hypotheses pertaining to the interrelationships of spiritual leadership, meaningful work, work engagement, and service recovery performance, hitherto no study has incorporated spiritual leadership, meaningful work, work engagement and service performance into an integrated framework. Thus, to better systematically determine the mediating effect between the above variables, to have more profound and comprehensive understanding of the psychological mechanism between spiritual leadership and service performance, and last but not least to more efficiently enhance the ecological validity of this research, this study firstly proposes the following hypothesis:

Hypothesis 9: Spiritual leadership is positively related to service performance via Meaningful work and work engagement.

3. Method

The materials, method and procedures performed in this study involving human participants conform to the 1964 Helsinki declaration and its later amendments or comparable ethical standards, and they were approved by the Human Research Ethics Committee of Shaanxi Normal University, and informed consent was obtained from all individual participants included in this study.

3.1 Participants

From Feb. 23rd, 2022 to June 30th, 2024, a total of 320 Chinese flight attendants from Tibet Airlines completed the self-reported measures online. On June 30th, 2024, a total of 313 valid questionnaires were obtained and accessed (effective rate of 97.8%) for research purposes. 75 participants were male and 238 participants were female, including 3 cabin managers, 62 pursers, 81 business\first class flight attendants, 156 economy class flight attendants and 11 flight attendants trainee. The flight attendants' age ranged from 21 to 50 years old (M = 27.66, SD = 4.85), total flight hours ranged from 110 to 26,000 h (M =

4502.90, SD = 4249.39) and their years of service for the cooperation from 1 year to 29 years (M = 5.96, SD = 4.87). All participants were given detailed information about the academic purpose of this study, and were assured their response would be confidential. Also, they were guaranteed verbally that their individual response would not be identified in the results, and would not have any influence on their future career, which was witnessed by their leaders from Tibet Airlines. They all voluntarily participated in this study and answered the questionnaires truthfully.

3.2 Measures

Referring to Brislin's [115] back-translation procedure, the original survey items were translated into Chinese and then back-translated into English. Furthermore, the reliability and validity of each measure were examined via confirmatory factor analysis (CFA)and internal consistency test. Detail for each measure is present below.

3.2.1 Spiritual Leadership

This variable was evaluated by using the spiritual leadership scale from Fry et al. [23], which is a 17-item scale. Sample items like "My organization's vision is clear and compelling to me" (vision), "I always do my best in my work because I have faith in my organization and its leaders" (Hope/faith), and "My organization really cares about its people" (Altruistic love). All items were measured using a 5- point Likert scale, ranging from 1 (strongly disagree) to 5 (strongly agree). A high score on the scale indicates a high level of spiritual leadership. The coefficient α for this scale in the present study was 0.973. Confirmatory factor analysis was conducted to analyze the structural validity of the constructs. The results in the present study showed a good fit with the data: $\chi 2$ /df = 2.357, GFI = 0.932, NFI = 0.972, TLI = 0.971, CFI = 0.983, RMR=0.031, RMSEA = 0.066.

3.2.2 Meaningful work

Meaningful work was assessed by using Work as Meaning Inventory (WAMI) scale from Steger et al. [44], which is a ten-item scale. Sample items like "I have found a meaningful career" (Positive meaning), "I view my work as contributing to my personal growth" (Meaning making through work), and "I know my work makes a positive difference in the world" (Greater good motivations). All items were measured using a 5- point Likert scale, ranging from 1 (strongly disagree) to 5 (strongly agree). The coefficient α for this scale in the present study was 0.924. Confirmatory factor analysis was conducted to analyze the structural validity of the constructs. The results in the present study showed a good fit with the data: $\chi 2$ /df = 1.612, GFI = 0.975, NFI = 0.984, TLI = 0.989, CFI = 0.994, RMR=0.020, RMSEA = 0.044.

3.2.3 Work engagement

Work engagement was assessed by using UWES-9 from Schaufeli et al. [116], which is a nine-item scale. Sample items like "At my work, I feel bursting with energy" (Vigor), "I am enthusiastic about my job" (Dedication) and "I am immersed in my work" (Absorption). All items were measured using a 7- point Likert scale, ranging from 0 (never) to 6 (Always). A high score on the scale indicates a high level of work engagement. The coefficient α for this scale in the present study was 0.966. Confirmatory factor analysis was conducted to analyze the structural validity of the constructs. The results in the present study showed a good fit with the data: $\chi 2$ /df = 1.677, GFI = 0.979, NFI = 0.991, TLI = 0.993, CFI = 0.996, RMR=0.022, RMSEA = 0.047.

3.2.4 Service performance

To evaluate Service performance, a scale with four items from Liao and Chuang's [17] was adopted. Sample items like "I am friendly and helpful to customers", "I am approaching customers quickly" and "I am asking good questions and listening to find out what a customer wants". All Items measured on a scale ranging from 1 (strongly disagree) to 5 (strongly agree). A high score on the scale indicates better service performance. The coefficient α for this scale

in the present study was 0.948. Confirmatory factor analysis was conducted to analyze the structural validity of the constructs. The results in the present study showed a good fit with the data: $\chi 2$ /df = 2.149, GFI = 0.993, NFI = 0.997, TLI = 0.995, CFI = 0.998, RMR=0.004, RMSEA = 0.061.

3.2.5. Control variables

Prior studies on flight attendants' service performance suggested that their service performance standards vary and are assessed on a cabin-by-cabin basis (e.g. first class, business class and economy class). Cabin service is normally affected by security or safety pressure, and flight attendants on different position undertake different security and safety tasks (Diane et al., 2013). Thus, to avoid confounding effects and enhance the accuracy of the results, we take flight attendant's position and demographic variables (e.g. Age, marital status, Education) as control variables when testing the hypotheses, which is also congruent with other researches in the context of flight attendants' service performance [7, 12].

3.3 Statistics analysis

SPSS 22.0 with PROCESS 3.3 and Amos 24.0 were used in analysis. First, the Cronbach's α coefficient of each measure was calculated by SPSS for evaluating internal consistency. Then the confirmatory factor analysis (CFA) was excuted by Amos to varify the validity of each measure and to exclude the influence of common-method bias, referring to the fit indices of the model to the data recommended by Hu and Bentler [117]. Next, the descriptive statistics calculation and correlations analysis were completed by SPSS. Last, Model 6 of the PROCESS with SPSS was employed to verify the mediating effect of meaningful work, and work engagement. The bias-corrected percentile bootstrap method [118, 119] was used in this study to assess the mediation effect, which is that a significant mediating effect was indicated if bootstrap confidence interval (CI) does not include zero based on 5000 random samples [120, 121].

4. Results

4.1 Common-method bias test

To reduce common-method biases, all the data in this research was collected based on flight attendants' anonymous and voluntary self-reports at different time and places, and all the participants are guaranteed that their responses were confidential. To eliminate the possibility of common method bias, the Harman's single-factor test involving loading all latent variable indicators on a single latent factor in a confirmatory factor analysis (CFA) as a post-hoc statistical analysis [122] was performed via using Amos 24.0. The results demonstrated that the single factor model fit poorly to the data: $\chi 2$ /df = 8.494, GFI = 0.362, NFI = 0.606, TLI = 0.614, CFI = 0.634, RMR=0.125, RMSEA = 0.155, which provides further evidence that common method bias did not affect the significance of the results (Malhotra et al., 2006).

4.2 Preliminary analysis

Descriptive statistics analysis and correlations analysis for all variables are shown in Table 1, and the correlation coefficients of the main variables were statistically significant. For example, Spiritual leadership has positive relationship with meaningful work, work engagement and service performance. Meaningful work is positively related with work engagement and service performance. Work engagement is positively correlated with service performance. Thus, according to Baron and Kenny [123] and Mackinnon [124], above significant correlations confirm the relationships between the variables discussed in the hypothesis, and meet the requirements to conduct the mediation effect test.

Table 1. Descriptive statistics and correlations of the main variables (*N* = 313).

	Mean	SD	1	2	3	4	5	6	7
1. Age	27.66	4.85	-	-	-	-	-	-	-
2. Years of Service	5.96	4.87	0.93^{**}	-	-	-	-	-	-

3. Flight time	4502.90	4249.39	0.88**	0.95**	-	-	-	-	-
4. Spiritual leadership[a]	3.81	0.83	0.15**	0.14*	0.11	-	-	-	-
5. Meaningful work[a]	3.74	0.72	0.22**	0.19**	0.19**	0.82**	-	-	-
6. Work engagement[b]	5.03	1.20	0.24**	0.20**	0.18**	0.70**	0.68**	-	-
7. Service performance[a]	4.31	0.74	0.21**	0.21**	0.19**	0.61**	0.62**	0.54**	-

Notes: **p* < 0.05; ***p* < 0.01. aRange 1–5; bRange 1–6.

4.3 Mediation effects analysis

After introducing four types of control variables mentioned above, a test for mediating effects was performed by using PROCESS 3.3. The analysis results are present in Table 2 and Figure 2, and Table 2 is designed and categorized based on the same outcome variable. As model 1 shows, spiritual leadership is positively related with meaningful work (b=0.699, p<0.001), supporting Hypothesis 2. In model 2, spiritual leadership is positively associated with flight attendants' work engagement (b=0.649, p<0.001), which supports Hypothesis 6, and meaningful work positively affect flight attendant's work engagement (b=0.486, p<0.001), supporting Hypothesis 8. In model 3, spiritual leadership (b=0.227, p<0.01), meaningful work (b=0.317, p<0.001) and work engagement (b=0.081, p<0.05) all have positively influence on flight attendants' service performance, which supports Hypothesis 1, Hypothesis 3 and Hypothesis 5 respectively. Up to now, Hypothesis 1, Hypothesis 2, Hypothesis 3, Hypothesis 5, Hypothesis 6 and Hypothesis 8 are proved to be true. It should be noted that, in model 3, the calculated effect of spiritual leadership on service performance is direct effect, while in model 4, the calculated effect of spiritual leadership on service performance is total effect (b=0.529, p<0.001).

Fig. 2. The chain mediating effect of meaningful work and work engagement.

Note: *p<0.05, **p<0.01 and ***p<0.001.

Table 2. Regression results of the chain mediating effects model (*n*=313).

Outcome variable	Predictive variable	R^2	F	b	SEs	t	95% LLCI	95% ULCI
Model 1: Meaningful Work	Spiritual Leadership	0.680	130.426***	0.699***	0.029	24.192	0.642	0.756
Model 2: Work Engagement	Spiritual Leadership	0.541	60.007***	0.649***	0.098	6.632	0.457	0.842
	Meaningful Work			0.486***	0.113	4.288	0.263	0.709
Model 3: Service Performance	Spiritual Leadership	0.442	34.556***	0.227**	0.071	3.194	0.087	0.367
	Meaningful Work			0.317***	0.079	4.005	0.161	0.473
	Work Engagement			0.081*	0.039	2.089	0.005	0.157
Model 4: Service Performance	Spiritual Leadership	0.395	40.094***	0.529***	0.040	13.076	0.450	0.609

Note: 1. Control variables of the regression model include age, gender, flight time, years of service.

2. LLCI = Lower Limit Confidence Intervals; ULCI = Upper Limit Confidence Intervals.

3. *p<0.05, **p<0.01 and ***p<0.001.

As present in Table 3, the results of the bootstrap analysis (a bootstrap sample of 5000 was

specified) for meaningful work and work engagement separately of spiritual leadership on service performance, which are path 1 and path 3 in Table 3, do not include zero. Thus, Hypothesis 4 was supported because meaningful work mediated the relationship between spiritual leadership and service performance, and Hypothesis 7 was supported because work engagement mediated the relationship between spiritual leadership and service performance. In addition, meaningful work and work engagement played a chain mediation effect on relationship between spiritual leadership and service performance, considering path 2 in Table 3 do not include zero either. Hence, Hypothesis 9 was supported.

Table 3. Results and comparison of chain mediating effect (n=313)

Path: Independent Variable-Mediated Variable-Dependent Variable	95% LLCI	Estimated	95% ULCI
Path1: SL-MW-SP	0.080	0.222	0.344
Path2: SL-MW-WE-SP	0.006	0.028	0.066
Path3: SL-WE-SP	0.010	0.053	0.119

Note: 1. SP = Spiritual Leadership; MW = Meaningful Work; WE = Work Engagement; SP = Service Performance.

1. LLCI = Lower Limit Confidence Intervals; ULCI = Upper Limit Confidence Intervals.

In general, the results of the mediating effects analysis showed that there are four tenable paths that spiritual leadership could walk through to influence flight attendants' service performance (see Figure 2): (1) Spiritual Leadership - Meaningful Work - Service Performance; (2) Spiritual Leadership - Meaningful Work - Work Engagement - Service Performance; (3) Spiritual Leadership - Work Engagement - Service Performance; (4) Spiritual Leadership - Service Performance.

5. Discussion

5.1 Theoretical implications

First, previous empirical evidence lacks either depth or width of research on leadership and service related work outcome in flight attendants. Among very limited empirical evidence, Pio [57] and Yang et al. [36] have discussed the relationship between spiritual leadership and job performance. Meanwhile, Ali et al. [28] confirmed spiritual leadership has influence on airline pilots' safety behavior. In addition, Karatepe and Talebzadeh [20] proved psychological capital and work engagement mediated the relationship between servant leadership and pursers' service recovery performance. Besides, Tsai and Su [125] demonstrated job satisfaction mediated the relationship between leadership, e.g., transformational leadership and transactional leadership, and service-oriented organizational citizenship behaviors in flight attendants. Although leadership and flight attendants' service performance are both critical issues in survival, prosper and management of airlines [22, 53], the existing research is not adequate. On the one hand, previous research on flight attendants' job performance, more specifically saying, service performance, is confined to individual perspective, such as personality [16, 17], self-efficacy [18], psychological capital [19], psychological contract [49], burnout [12], few took organizational factors into consideration. On the other hand, previous limited literature provided little understanding of how and when spiritual leadership promote job performance [36]. However, this investigation deeply penetrated into the psychological mechanism between spiritual leadership and flight attendants' service performance, which not only contributed to the existing literature of leadership theory and boosted the diversification of the research on service performance, but also originally provided insight into the systematical understanding of specific motivational process that spiritual leadership exploit to influence service performance in the perspective of organizational psychology, positive psychology and occupational psychology.

Second, as exhibited in the results, we found that spiritual leadership, meaningful work and work engagement all have a positive impact on flight attendants' service performance in different ways. Specifically saying, spiritual leadership, meaningful work and work engagement can directly and positively affect flight attendants' service performance respectively. Meanwhile, spiritual leadership can positively influence meaningful work and work engagement, and indirectly influence flight attendants' service performance. Therefore, meaningful work and work engagement produce a partially mediating effect between spiritual leadership and service performance. Furthermore, through multiple pathways, such as spiritual leadership - meaningful work - service performance (SL-MW-SP), spiritual leadership - meaningful work - work engagement - service performance (SL-MW-WE-SP) and spiritual leadership - work engagement - service performance (SL-WE-SP), meaningful work and work engagement not merely play a simple mediating role between spiritual leadership and service performance, but jointly play a chain mediating role. Hence, meaningful work and work engagement are two important factors when analyzing the psychological mechanism between spiritual leadership and flight attendants' service performance. Previous studies have shown that meaningful work act as a mediator between transformational leadership and work engagement [46] and employees' psychological well-being [126], but whether meaningful work could mediate between spiritual leadership and service performance has not been examined until now. This study differs from existing literature of leadership and service performance in that, by applying Job Characteristics Theory [30, 79], it indicates that spiritual leadership promotes flight attendants' three critical psychological states, including work meaningfulness, thus further resulting in quality service performance [31, 32]. At the same time, our results revealed that work engagement appears to mediate the relationship between spiritual leadership and service performance. Drawing upon Job Demands-Resources (JD-R) theory [100], job resources would become salient and gain motivational potential when employees confront with

high job demands (Bakker, 2010), and thus work engagement being further affected. This enriches our understanding of how spiritual leadership influences service performance, which echoes previous research on work engagement and work performance of flight attendants [18].

Lastly, Our results extends the empirical research of positive psychology and further advances the understanding of psychological mechanism between spiritual leadership and service performance. Our research found that meaningful work and work engagement, core dimensions of happiness and well-being [127], can jointly play a chain mediating role between spiritual leadership and service performance, rather than just a simple mediating role. This finding is consistent with the results of prior studies of chain mediating role of meaningful work and work engagement [48, 112, 128], but under the theoretical framework of JD-R theory, the underlying motivational process between spiritual leadership and service performance is emerging. Based on JD-R theory, job resources and personal resources would lead to the satisfaction of psychological needs (e.g., need for meaningfulness, belongingness, competence) that in turn result in work engagement and flight attendants' service performance [45, 100, 104]. To our knowledge, this is the first study to prove the existence of the path of spiritual leadership-meaningful work - work engagement - service performance in the context of civil aviation.

Overall, this study demonstrates the importance of spiritual leadership, meaningful work, work engagement and service performance, highlights its potential usefulness in airlines management, and fulfills the theoretical and empirical research of organizational psychology, positive psychology and occupational psychology.

5.2 Managerial implications

The application of positive psychology to the context of work environment has attracted large interest within both academic and practitioner domains over the past decade. In the perspective of practitioners, there has been a proliferation of organizational development, leadership development, team development and coaching programs, initiatives, and

interventions that have positive psychological principles at their core [29, 129-131]. This study provides some feasible managerial practices for promoting flight attendants' service performance to prepare for welcoming increasing demand of air transportation and to help airlines prosper.

First, our findings suggest that spiritual leadership has a significant positive effect on flight attendants' service performance, which is consistent with the researches conducted by Pio's [57] and Yang et al. [36]. Thus, one practical implication of our study is that it shows the importance of acknowledging the significance of spiritual leadership in promoting flight attendants' service performance. Many successful organizations such as Ford Motor Company, AT&T, the World Bank, Apple and Chase Bank have developed plans and programs to bring spiritual leadership to the work environment more or less [68, 132]. Specifically, leaders and managers of airlines should possess spiritual leadership competencies, create a clear and compelling vision of where the airlines wants to be in the near and distant future, emphasize the importance of service performance during the flight, and inspire flight attendants' best performance [74]. Meanwhile, airline leaders should shape a working environment based on the altruistic love, demonstrate spiritual behaviors personally, help flight attendants overcome job-related difficulties and ensure positive human health, whereby both leaders and employees have a sense of membership, belongingness and feel understood and appreciated [28, 35, 64]. In this case, leaders and managers of airlines may be able to boost high organizational commitment and productivity among flight attendants, and thus result in flight attendants' quality of service performance in turn.

Second, the current research highlights that meaningful work and work engagement not merely play a simple mediating role separately between spiritual leadership and service performance, but jointly play a chain mediating role, which provides airline leaders a new entry point to foster a higher level of flight attendants' service performance. For example, In the daily

management of airlines, leaders of cabin service department should pay more attention to intrinsic and extrinsic motivation, integrating spirituality into working environment and organization culture because employees seek to improve the quality of their working life by finding meaning within their work and achieve life goals beyond material and earnings [133]. In addition, leaders of cabin service department could periodically host online or offline forum sharing the feeling that flight attendants confronted with during the daily flight task, taking care of flight attends' moral value and mental feeling and offering communication transparency between leaders and employees [134]. Especially when investigating the case of passengers' complaints, developing open communication between leaders and flight attendants is more important [135] than giving punishment, because mere punishment would kill flight attendants' work engagement. Furthermore, series of short movies about the job meaning of flight attendants could be broadcast, showing what they bring to passenger's journey and what difference they make to the world, when hosting seminars about skill promotion. All of these options would help to promote the flight attendants' service performance.

Last, the above practical advisories were generated according to the findings of this research. From point of adopting spiritual leadership in daily management to instilling work meaningfulness and promotion of work engagement, the managerial tactics and strategies could be used alone and corporately, and would have a positive effect on promoting flight attendants' service performance.

5.3 Limitations and future research directions

Although this research has the above contributions both theoretically and practically, there are some limitations that can be discussed in future study. First of all, this research used a cross-sectional design [120, 136], which did not allow for the examination of changes in flight attendants' psychological state and perceived leadership with time change. Thus, the predictive value of the research data may be limited. Future research could adopt a longitudinal design to

investigate the relationship between these variables and to develop causality. Second, the data was collected from flight attendants serving for Tibet Airlines and its branches. Hence, it is not clear whether the conclusions of this research are applicable to other flight attendants serving for different airlines, such as Air China, China Southern Airlines, Tibet Airlines, or even other service-related occupations in civil aviation, such as ground service employees. Last, our research found that meaningful work and work engagement are two important psychological links between spiritual leadership and service performance. However, other possible psychological mechanisms that mediate the relationship between spiritual leadership and flight attendants' service performance could be explored in the future.

6. Conclusion

In general, this research demonstrated the positive relationship between spiritual leadership and flight attendants' service performance in a multidisciplinary perspective, i.e., organization psychology, positive psychology and vocational psychology. Drawing upon job characteristic theory, this study demonstrated the mediating role of meaningful work in interpretation of positive relationship between spiritual leadership and service performance. Meanwhile, under the framework of JD-R theory, this research revealed that not only work engagement mediate the positive relationship between spiritual leadership and service performance but also meaningful work and work engagement can jointly play a chain mediating role between spiritual leadership and service performance. By examining whether and how spiritual leadership influences flight attendants' service performance, this research contributes to the existing theory of spiritual leadership, boost the diversification of research on service performance, offer the empirical evidence to the application of job characteristic theory and JD-R theory and apply organizational psychology, positive psychology and vocational psychology into the context of airline management. Importantly, it provides practical implications regarding how to promote flight attendants' service performance based on psychological mechanism.

List of abbreviations

IATA: International Air Transport Association

JCT: Job Characteristics Theory

JD-R: Job Demands-Resources

CFA: confirmatory factor analysis

Declarations

Ethics approval and consent to participate

This study was conducted in accordance with the Declaration of Helsinki and was approved by the Human Research Ethics Committee of Shaanxi Normal University, and informed consent was obtained from all individual participants included in this study.

Consent for publication

Not applicable.

Availability of data and materials

The datasets used and/or analysed during the current study are available from the corresponding author on reasonable request.

Competing interests

The authors declare that they have no competing interests.

Funding

This work was supported by Key Research and Development Program of Shaanxi (2021SF-481) ,National Natural Science Foundation of China（32000753）

Authors' contributions

Lei Du: Writing-original manuscript, writing-review or editing, conceptualization, data management, methodology, formal analysis, Validation. Ying Li, Yangliang Wu, Ming Ji, Jun Zhang, Zixian Li, Mingliang Li: Writing Reviewing or editing, conceptualization, data

management, methodology, supervision. Tao Wen，Fei Gu, Chenguang Pan, Yuan Li, Xuqun You, Saifang Liu: data management. All authors contributed to this study and approved the final submitted manuscript.

Acknowledgements

The authors would like to appreciate all the flight attendants from Tibet Airlines Co., Ltd. and its branches who answered our questionnaire. Meanwhile, the authors would like to appreciate the leaders from cabin service department of Tibet Airlines for their strong support and assistance.

References

1. Damos DL, Boyett KS, Gibbs P. Safety versus passenger service: The flight attendants' dilemma. The international journal of aviation psychology. 2013; 23(2):91-112.
2. Chen AH, Peng N, Hackley C. Evaluating service marketing in airline industry and its influence on student passengers' purchasing behavior using Taipei–London route as an example. Journal of Travel & Tourism Marketing. 2008; 25(2):149-60.
3. International Air Transport Association. Annual Review 2020. In *76th Annual General Meeting*. Amsterdam, Netherlands; 2020.
4. International Air Transport Association. Annual Review 2022. In *78th Annual General Meeting and World Air Transport Summit*. Doha, Qatar; 2022.
5. Akamavi RK, Mohamed E, Pellmann K, Xu Y. Key determinants of passenger loyalty in the low-cost airline business. Tourism Management. 2015; 46:528-45.
6. Han H, Hyun SS, Kim W. In-flight service performance and passenger loyalty: A cross-national (China/Korea) study of travelers using low-cost carriers. Journal of Travel & Tourism Marketing. 2014; 31(5):589-609.
7. Karatepe OM, Vatankhah S. The effects of high-performance work practices and job embeddedness on flight attendants' performance outcomes. Journal of Air Transport Management. 2014; 37:27-35.
8. Karatepe OM, Eslamlou A. Outcomes of job crafting among flight attendants. Journal of Air Transport Management. 2017; 62:34-43.
9. Shen C, Yahya Y. The impact of service quality and price on passengers' loyalty towards low-cost airlines: The Southeast Asia perspective. Journal of Air Transport Management. 2021; 91:101966.
10. Chang C-P, Ju-Mei C. Flight attendants' emotional labor and exhaustion in the Taiwanese airline industry. Journal of Service Science and Management. 2009;

2(4):305-11.

11. Yeh C-W. Service climate, professional commitment and job performance of flight attendants in Taiwan. Journal of Air Transport Management. 2009; 15(5):259-60.

12. Chen C-F, Kao Y-L. Moderating effects of work engagement and job tenure on burnout–performance among flight attendants. Journal of Air Transport Management. 2012; 25:61-63.

13. Hsu Y-L, Liu T-C. Structuring risk factors related to airline cabin safety. Journal of air transport management. 2012; 20:54-56.

14. Kim Y, Back K-J. Antecedents and consequences of flight attendants' job satisfaction. The Service Industries Journal. 2012; 32(16):2565-84.

15. Ng SI, Sambasivan M, Zubaidah S. Antecedents and outcomes of flight attendants’ job satisfaction. Journal of Air Transport Management. 2011; 17(5):309-13.

16. Chen C-F, Kao Y-L. Investigating the moderating effects of service climate on personality, motivation, social support, and performance among flight attendants. Tourism Management. 2014; 44:58-66.

17. Liao H, Chuang A. A multilevel investigation of factors influencing employee service performance and customer outcomes. Academy of Management journal. 2004; 47(1):41-58.

18. Xanthopoulou D, Baker AB, Heuven E, Demerouti E, Schaufeli WB. Working in the sky: a diary study on work engagement among flight attendants. Journal of occupational health psychology. 2008; 13(4):345.

19. Cheng T-M, Hong C-Y, Yang B-C. Examining the moderating effects of service climate on psychological capital, work engagement, and service behavior among flight attendants. Journal of Air Transport Management. 2018; 67:94-102.

20. Karatepe OM, Talebzadeh N. An empirical investigation of psychological capital among

flight attendants. Journal of Air Transport Management. 2016; 55:193-202.

21. Wu H-C, Cheng C-C. A hierarchical model of service quality in the airline industry. Journal of Hospitality and Tourism Management. 2013; 20:13-22.

22. Hogan R, Kaiser RB. What we know about leadership. Review of general psychology. 2005; 9(2):169-80.

23. Fry LW, Vitucci S, Cedillo M. Spiritual leadership and army transformation: Theory, measurement, and establishing a baseline. The leadership quarterly. 2005; 16(5):835-62.

24. Liden RC, Wayne SJ, Zhao H, Henderson D. Servant leadership: Development of a multidimensional measure and multi-level assessment. The leadership quarterly. 2008; 19(2):161-77.

25. Chughtai AA. Creating safer workplaces: The role of ethical leadership. Safety science. 2015; 73:92-98.

26. Bass BM, Avolio BJ, Jung DI, Berson Y. Predicting unit performance by assessing transformational and transactional leadership. Journal of applied psychology. 2003; 88(2):207.

27. Wang M, Guo T, Ni Y, Shang S, Tang Z. The effect of spiritual leadership on employee effectiveness: An intrinsic motivation perspective. Frontiers in psychology. 2019; 9:2627.

28. Ali M, Aziz S, Pham TN, Babalola MT, Usman M. A positive human health perspective on how spiritual leadership weaves its influence on employee safety performance: The role of harmonious safety passion. Safety science. 2020; 131:104923.

29. Albrecht SL. Work engagement and the positive power of meaningful work. In *Advances in positive organizational psychology.* Emerald Group Publishing Limited; 2013: 237-60

30. Hackman JR, Oldham GR. Motivation through the design of work: Test of a theory.

Organizational behavior and human performance. 1976; 16(2):250-79.

31. Allan BA, Duffy RD, Collisson B. Task significance and performance: Meaningfulness as a mediator. Journal of Career Assessment. 2018; 26(1):172-82.

32. Allan BA, Batz-Barbarich C, Sterling HM, Tay L. Outcomes of Meaningful Work: A Meta ‐ nalysis. Journal of Management Studies. 2019; 56(3):500-28.

33. Harris KJ, Kacmar KM, Zivnuska S. An investigation of abusive supervision as a predictor of performance and the meaning of work as a moderator of the relationship. The leadership quarterly. 2007; 18(3):252-63.

34. Steger MF, Dik BJ. Work as meaning: Individual and organizational benefits of engaging in meaningful work. In *Oxford handbook of positive psychology and work.* Edited by Linley PA, Harrington S, Garcea N. Oxford: Oxford University Press; 2009: 131-42

35. Fry LW, Nisiewicz MS. Maximizing the triple bottom line through spiritual leadership. Stanford University Press; 2013.

36. Yang F, Liu J, Wang Z, Zhang Y. Feeling energized: a multilevel model of spiritual leadership, leader integrity, relational energy, and job performance. Journal of Business Ethics. 2019; 158:983-97.

37. Martínez-Córcoles M, Gracia FJ, Tomás I, Peiró JM, Schöbel M. Empowering team leadership and safety performance in nuclear power plants: A multilevel approach. Safety science. 2013; 51(1):293-301.

38. Neal A, Griffin MA. A study of the lagged relationships among safety climate, safety motivation, safety behavior, and accidents at the individual and group levels. Journal of applied psychology. 2006; 91(4):946.

39. Schaufeli WB, Salanova M, González-Romá V, Bakker AB. The measurement of engagement and burnout: A two sample confirmatory factor analytic approach. Journal

of Happiness studies. 2002; 3:71-92.

40. De Simone S, Planta A, Cicotto G. The role of job satisfaction, work engagement, self-efficacy and agentic capacities on nurses' turnover intention and patient satisfaction. Applied Nursing Research. 2018; 39:130-40.

41. Britt TW, Adler AB, Bartone PT. Deriving benefits from stressful events: the role of engagement in meaningful work and hardiness. Journal of occupational health psychology. 2001; 6(1):53.

42. Kahn WA. Psychological conditions of personal engagement and disengagement at work. Academy of management journal. 1990; 33(4):692-724.

43. Maslach C, Leiter MP. The truth about burnout: How organizations cause personal stress and what to do about it. San Francisco, CA: Jossey-Bass; 2000.

44. Steger MF, Dik BJ, Duffy RD. Measuring meaningful work: The work and meaning inventory (WAMI). Journal of career Assessment. 2012; 20(3):322-37.

45. Bakker AB, Demerouti E. Towards a model of work engagement. Career development international. 2008; 13(3):209-23.

46. Yasin Ghadi M, Fernando M, Caputi P. Transformational leadership and work engagement: The mediating effect of meaning in work. Leadership & Organization Development Journal. 2013; 34(6):532-50.

47. May DR, Gilson RL, Harter LM. The psychological conditions of meaningfulness, safety and availability and the engagement of the human spirit at work. Journal of occupational and organizational psychology. 2004; 77(1):11-37.

48. Van Wingerden J, Poell RF. Meaningful work and resilience among teachers: The mediating role of work engagement and job crafting. PloS one. 2019; 14(9):e0222518.

49. Yeh C-W. Relationships among service climate, psychological contract, work engagement and service performance. Journal of Air Transport Management. 2012;

25:67-70.
50. Fu Y-K. The influence of internal marketing by airlines on customer-oriented behavior: A test of the mediating effect of emotional labor. Journal of Air Transport Management. 2013; 32:49-57.
51. Morgan M, Nickson D. Uncivil aviation: A review of the air rage phenomenon. International Journal of Tourism Research. 2001; 3(6):443-57.
52. Hvass KA, Torfadóttir E. Spatially dispersed employee recovery: an airline case study. Journal of air transport management. 2014; 34:65-69.
53. Lee C, An M, Noh Y. The effects of emotional display rules on flight attendants' emotional labor strategy, job burnout and performance. Service Business. 2015; 9:409-25.
54. Liang S-C, Hsieh A-T. Individual's perception of career development and job burnout among flight attendants in Taiwan. The International Journal of Aviation Psychology. 2005; 15(2):119-34.
55. Rhoden S, Ralston R, Ineson EM. Cabin crew training to control disruptive airline passenger behavior: A cause for tourism concern? Tourism Management. 2008; 29(3):538-47.
56. Gabbott M, Tsarenko Y, Mok WH. Emotional intelligence as a moderator of coping strategies and service outcomes in circumstances of service failure. Journal of Service Research. 2011; 14(2):234-48.
57. Pio RJ. The mediation effect of quality of worklife and job satisfaction in the relationship between spiritual leadership to employee performance. International Journal of Law and Management. 2022; 64(1):1-17.
58. Krishnakumar S, Houghton JD, Neck CP, Ellison CN. The "good" and the "bad" of spiritual leadership. Journal of Management, Spirituality & Religion. 2015; 12(1):17-

37.

59. Barling J, Loughlin C, Kelloway EK. Development and test of a model linking safety-specific transformational leadership and occupational safety. Journal of applied psychology. 2002; 87(3):488.

60. Turner N, Barling J, Epitropaki O, Butcher V, Milner C. Transformational leadership and moral reasoning. Journal of applied Psychology. 2002; 87(2):304.

61. Lu C-S, Yang C-S. Safety leadership and safety behavior in container terminal operations. Safety science. 2010; 48(2):123-34.

62. Giacalone R, Jurkiewicz CL. Toward a science of workplace spirituality. In *Handbook of Workplace Spirituality and Organizational Performance.* Edited by Giacalone R, Jurkiewicz C. Armonk, NY: ME Sharpe; 2003

63. Chen C-Y, Yang C-F. The impact of spiritual leadership on organizational citizenship behavior: A multi-sample analysis. Journal of business ethics. 2012; 105:107-14.

64. Fry LW, Latham JR, Clinebell SK, Krahnke K. Spiritual leadership as a model for performance excellence: a study of Baldrige award recipients. Journal of Management, Spirituality & Religion. 2017; 14(1):22-47.

65. Jurkiewicz CL, Giacalone RA. A values framework for measuring the impact of workplace spirituality on organizational performance. Journal of business ethics. 2004; 49:129-42.

66. Fry L, Matherly L. Workplace spirituality, spiritual leadership and performance excellence. Encyclopedia of Industrial/Organizational Psychology (Sage Publications, San Francisco). 2007.

67. Crossman J. Conceptualising spiritual leadership in secular organizational contexts and its relation to transformational, servant and environmental leadership. Leadership & Organization Development Journal. 2010; 31(7):596-608.

68. Salehzadeh R, Khazaei Pool J, Kia Lashaki J, Dolati H, Balouei Jamkhaneh H. Studying the effect of spiritual leadership on organizational performance: an empirical study in hotel industry. International journal of culture, tourism and hospitality Research. 2015; 9(3):346-59.
69. Chi N-W, Grandey AA. Emotional labor predicts service performance depending on activation and inhibition regulatory fit. Journal of Management. 2019; 45(2):673-700.
70. Shin Y, Lee EJ, Hur W-M. Supervisor incivility, job insecurity, and service performance among flight attendants: the buffering role of co-worker support. Current Issues in Tourism. 2022; 25(6):901-18.
71. Van Gelderen BR, Konijn EA, Bakker AB. Emotional labor among police officers: A diary study relating strain, emotional labor, and service performance. The International Journal of Human Resource Management. 2017; 28(6):852-79.
72. Daft RL, Lengel RH. Fusion leadership: Unlocking the subtle forces that change people and organizations. Berrett-Koehler Publishers; 1998.
73. Kotter JP. Leading change. Boston, MA: Harvard business press; 1996.
74. Fry LW. Toward a theory of spiritual leadership. The leadership quarterly. 2003; 14(6):693-727.
75. Fry LW, Cohen MP. Spiritual leadership as a paradigm for organizational transformation and recovery from extended work hours cultures. Journal of business ethics. 2009; 84:265-78.
76. Duchon D, Plowman DA. Nurturing the spirit at work: Impact on work unit performance. The leadership quarterly. 2005; 16(5):807-33.
77. Pfeffer J. Business and the spirit: Management practices that sustain values. In *Handbook of workplace spirituality and organizational performance.* M. E. Sharp, New York, NY: Routledge; 2003: 43-59

78. Lips-Wiersma M, Wright S, Dik B. Meaningful work: differences among blue-, pink-, and white-collar occupations. Career Development International. 2016; 21(5):534-51.

79. Oldham GR, Hackman JR. Not what it was and not what it will be: The future of job design research. Journal of organizational behavior. 2010; 31(2-3):463-79.

80. Chalofsky N. An emerging construct for meaningful work. Human Resource Development International. 2003; 6(1):69-83.

81. Lips-Wiersma M. The influence of spiritual "meaning-making" on career behavior. Journal of Management Development. 2002; 21(7):497-520.

82. Garcia-Zamor JC. Workplace spirituality and organizational performance. Public administration review. 2003; 63(3):355-63.

83. Kirkpatrick SA, Locke EA. Direct and indirect effects of three core charismatic leadership components on performance and attitudes. Journal of applied psychology. 1996; 81(1):36.

84. Piccolo RF, Colquitt JA. Transformational leadership and job behaviors: The mediating role of core job characteristics. Academy of Management journal. 2006; 49(2):327-40.

85. Bakker AB, Schaufeli WB, Leiter MP, Taris TW. Work engagement: An emerging concept in occupational health psychology. Work & stress. 2008; 22(3):187-200.

86. Christian MS, Garza AS, Slaughter JE. Work engagement: A quantitative review and test of its relations with task and contextual performance. Personnel psychology. 2011; 64(1):89-136.

87. Halbesleben JR. A meta-analysis of work engagement: Relationships with burnout, demands, resources, and consequences. Work engagement: A handbook of essential theory and research. 2010; 8(1):102-17.

88. Knight C, Patterson M, Dawson J. Building work engagement: A systematic review and meta-analysis investigating the effectiveness of work engagement interventions. Journal

of organizational behavior. 2017; 38(6):792-812.

89. Bakker AB, Albrecht S. Work engagement: current trends. Career development international. 2018; 23(1):4-11.

90. Ployhart RE, Turner SF. Organizational adaptability. In *Individual Adaptability to Changes at Work.* New York, NY: Routledge; 2014: 73-91

91. Bakker AB. An evidence-based model of work engagement. Current directions in psychological science. 2011; 20(4):265-69.

92. Chaurasia S, Shukla A. Psychological capital, LMX, employee engagement & work role performance. The Indian Journal of Industrial Relations. 2014:342-56.

93. Siddiqi MA. Work engagement and job crafting of service employees influencing customer outcomes. Vikalpa. 2015; 40(3):277-92.

94. Guest D. Employee engagement: a sceptical analysis. Journal of Organizational Effectiveness: People and Performance. 2014; 1(2):141-56.

95. Schaufeli HB, Salanova M. How to improve work engagement? In *The handbook of employee engagement: Perspectives, issues, research and practice.* Edited by Albrecht S. Cheltenham, UK: Edward Elgar Publishing Northampton; 2010: 399-415

96. Bakker AB, Albrecht SL, Leiter MP. Key questions regarding work engagement. European journal of work and organizational psychology. 2011; 20(1):4-28.

97. Lai F-Y, Tang H-C, Lu S-C, Lee Y-C, Lin C-C. Transformational leadership and job performance: The mediating role of work engagement. Sage Open. 2020; 10(1):2158244019899085.

98. Tims M, Bakker AB, Xanthopoulou D. Do transformational leaders enhance their followers' daily work engagement? The leadership quarterly. 2011; 22(1):121-31.

99. Vincent-Höper S, Muser C, Janneck M. Transformational leadership, work engagement, and occupational success. Career development international. 2012; 17(7):663-82.

100. Bakker AB, Demerouti E. The job demands-resources model: State of the art. Journal of managerial psychology. 2007; 22(3):309-28.

101. Demerouti E, Bakker AB, Nachreiner F, Schaufeli WB. The job demands-resources model of burnout. Journal of Applied psychology. 2001; 86(3):499.

102. Steger MF, Littman-Ovadia H, Miller M, Menger L, Rothmann S. Engaging in work even when it is meaningless: Positive affective disposition and meaningful work interact in relation to work engagement. Journal of Career Assessment. 2013; 21(2):348-61.

103. Xanthopoulou D, Bakker AB, Demerouti E, Schaufeli WB. The role of personal resources in the job demands-resources model. International journal of stress management. 2007; 14(2):121.

104. Bakker AB. Engagement and "job crafting": Engaged employees create their own great place to work. In *Handbook of employee engagement.* Edward Elgar Publishing; 2010

105. Luthans F, Youssef CM, Avolio BJ. Psychological capital: Developing the human competitive edge. Oxford: Oxford university press; 2007.

106. Xanthopoulou D, Bakker AB, Demerouti E, Schaufeli WB. Reciprocal relationships between job resources, personal resources, and work engagement. Journal of Vocational behavior. 2009; 74(3):235-44.

107. Schaufeli WB, Bakker AB. Job demands, job resources, and their relationship with burnout and engagement: A multi-sample study. Journal of Organizational Behavior: The International Journal of Industrial, Occupational and Organizational Psychology and Behavior. 2004; 25(3):293-315.

108. Karatepe OM. Hope, work engagement, and organizationally valued performance outcomes: An empirical study in the hotel industry. Journal of Hospitality Marketing & Management. 2014; 23(6):678-98.

109. Albrecht SL, Su MJ. Job resources and employee engagement in a Chinese context: the

mediating role of job meaningfulness, felt obligation and positive mood. International Journal of Business and Emerging Markets. 2012; 4(4):277-92.

110. Stairs M, Galpin M. Positive engagement: From employee engagement to workplace happiness. Oxford: Oxford University Press; 2009.

111. Williamson JC, Geldenhuys M. Positive work experiences and life satisfaction: The moderating role of gender. Journal of Psychology in Africa. 2014; 24(4):315-20.

112. Van Wingerden J, Van der Stoep J. The motivational potential of meaningful work: Relationships with strengths use, work engagement, and performance. PloS one. 2018; 13(6):e0197599.

113. Johnson MJ, Jiang L. Reaping the benefits of meaningful work: The mediating versus moderating role of work engagement. Stress and Health. 2017; 33(3):288-97.

114. Li Y, Liu Z, Qin K, Cui J, Zeng X, Ji M, Lan J, You X, Li Y. Organizational trust and safety operation behavior in airline pilots: The mediating effects of organizational identification and organizational commitment. Journal of Air Transport Management. 2021; 92:102018.

115. Brislin RW. Translation and content analysis of oral and written materials. Methodology. 1980:389-444.

116. Schaufeli WB, Bakker AB, Salanova M. The measurement of work engagement with a short questionnaire: A cross-national study. Educational and psychological measurement. 2006; 66(4):701-16.

117. Hu Lt, Bentler PM. Cutoff criteria for fit indexes in covariance structure analysis: Conventional criteria versus new alternatives. Structural equation modeling: a multidisciplinary journal. 1999; 6(1):1-55.

118. Edwards JR, Lambert LS. Methods for integrating moderation and mediation: a general analytical framework using moderated path analysis. Psychological methods. 2007;

12(1):1.

119. Preacher KJ, Rucker DD, Hayes AF. Addressing moderated mediation hypotheses: Theory, methods, and prescriptions. Multivariate behavioral research. 2007; 42(1):185-227.

120. Hayes AF. Introduction to mediation, moderation, and conditional process analysis: A regression-based approach. Guilford publications; 2018.

121. Wang Y, Xie T, Xu J. Family socioeconomic status and internalizing problem behavior among chinese adolescents: the chain mediation effect of academic performance and peer conflict. Frontiers in Psychology. 2022; 13:902545.

122. Podsakoff PM, MacKenzie SB, Lee J-Y, Podsakoff NP. Common method biases in behavioral research: a critical review of the literature and recommended remedies. Journal of applied psychology. 2003; 88(5):879.

123. Baron RM, Kenny DA. The moderator–mediator variable distinction in social psychological research: Conceptual, strategic, and statistical considerations. Journal of personality and social psychology. 1986; 51(6):1173.

124. MacKinnon JG. Bootstrap inference in econometrics. Canadian Journal of Economics/Revue canadienne d'économique. 2002; 35(4):615-45.

125. Tsai C-T, Su CS. Leadership, job satisfaction and service-oriented organizational citizenship behaviors in flight attendants. African Journal of Business Management. 2011; 5(5):1915-26.

126. Arnold KA, Turner N, Barling J, Kelloway EK, McKee MC. Transformational leadership and psychological well-being: the mediating role of meaningful work. Journal of occupational health psychology. 2007; 12(3):193.

127. Seligman M. Flourish: A visionary new understanding of happiness and well-being. New York, NY: Free Press; 2011.

128. Hulshof IL, Demerouti E, Le Blanc PM. Day-level job crafting and service-oriented task performance: The mediating role of meaningful work and work engagement. Career Development International. 2020; 25(4):355-71.
129. Keyes CL, Haidt J. Flourishing: Positive psychology and the life well-lived. Washington, DC: American Psychological Association; 2003.
130. Linley PA, Harrington S, Garcea N. Oxford handbook of positive psychology and work. Oxford University Press; 2010.
131. Luthans F. The need for and meaning of positive organizational behavior. Journal of Organizational Behavior: The International Journal of Industrial, Occupational and Organizational Psychology and Behavior. 2002; 23(6):695-706.
132. Petchsawang P, Duchon D. Measuring workplace spirituality in an Asian context. Human resource development international. 2009; 12(4):459-68.
133. Benefiel M. The second half of the journey: Spiritual leadership for organizational transformation. The leadership quarterly. 2005; 16(5):723-47.
134. Vogelgesang GR, Leroy H, Avolio BJ. The mediating effects of leader integrity with transparency in communication and work engagement/performance. The leadership quarterly. 2013; 24(3):405-13.
135. Miles EW, Patrick SL, King Jr WC. Job level as a systemic variable in predicting the relationship between supervisory communication and job satisfaction. Journal of Occupational and Organizational Psychology. 1996; 69(3):277-92.
136. Shrout PE. Commentary: Mediation analysis, causal process, and cross-sectional data. Multivariate Behavioral Research. 2011; 46(5):852-60.

Click here to access/download;Figure;Fig1.tif

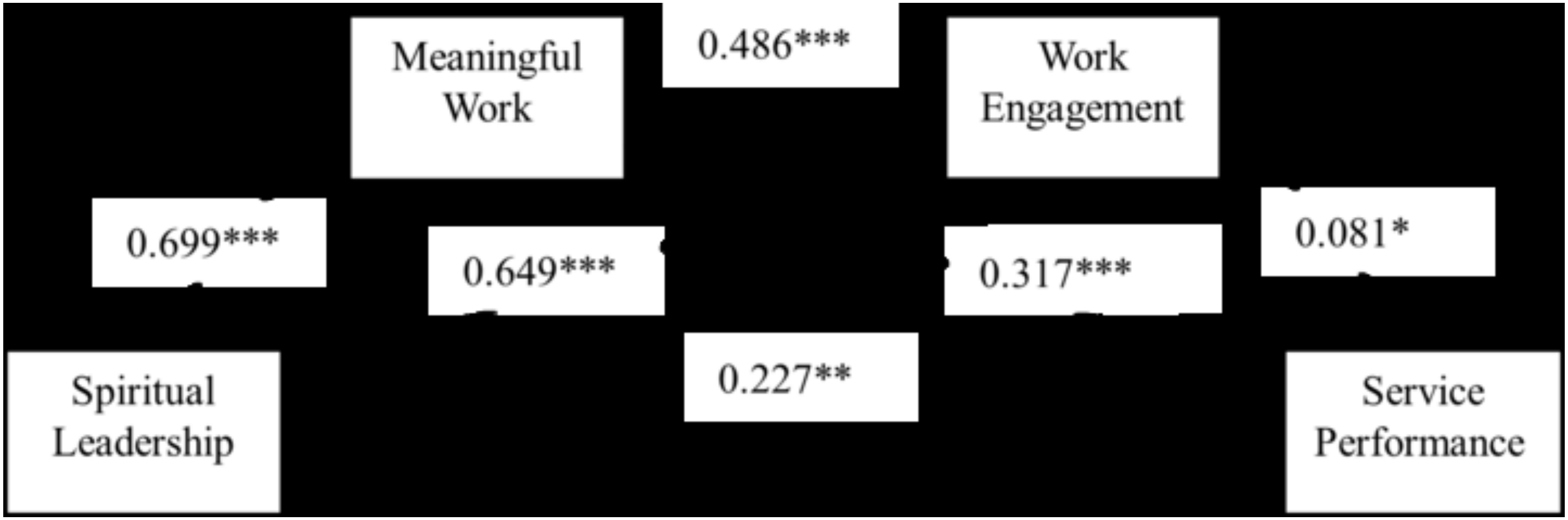

Click here to access/download;Figure;Fig2.tif

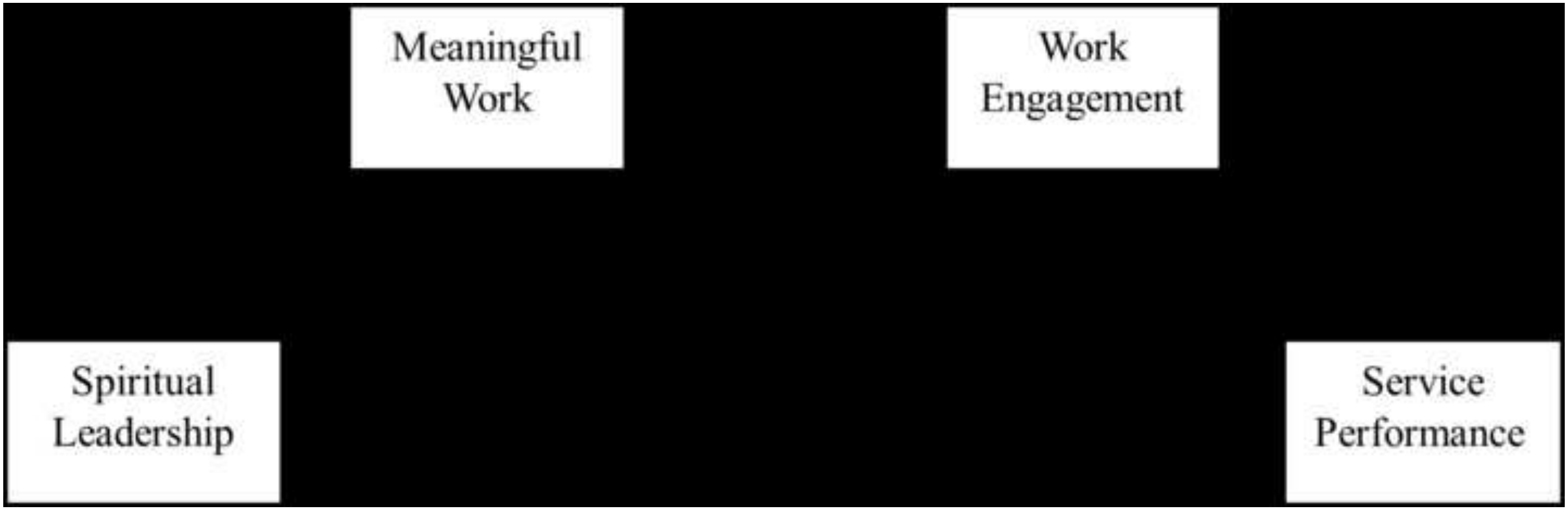

Click here to access/download
Supporting Information
the original ethics approval.pdf

Click here to access/download
Supporting Information
the translation ethics approval.pdf
