# Supplementary material for: Spiritual leadership and service performance among Chinese flight attendants: The mediating effects of meaningful work and work engagement
Source: PLoS One. 2026 May 21;21(5):e0324405. doi: 10.1371/journal.pone.0324405 (PMC13193339; doi:10.1371/journal.pone.0324405)
Supplement: Attachment — Submitted filename: Response to reviewers.docx [file pone.0324405.s003.docx]

Dear Professor Boshra A. Arnout and Reviewers,

Thank you for your letter and for the Reviewers’ comments concerning our manuscript entitled “Spiritual leadership and service performance among Chinese flight attendants: The mediating effects of meaningful work and work engagement” (ID: PONE-D-25-18162). Those comments are all valuable and very helpful for revising and improving our paper, as well as the important guiding significance to our research. We have studied the comments carefully and have made correction to meet with approval. The revised portions of the content are marked in the file of Revised Manuscript with Track Changes. Also, we have modified the wording mistakes in the entire manuscript. The main corrections in the paper and the detailed point-by-point responds to the reviewer’s comments are as follow:

**List of Responses**

**Responses to the reviewer # 1:**

**Reviewer #1:**  You need to correct all comments attached on the article.

**Comment #1 :** Abstract:where is the method of the study.

**Response:** Thank you very much for your comments. In response to your suggestions, we have rewritten the **Abstract** section and incorporated details of the study methodology into it. Specifically, revisions were made with attention to the following aspects: 1. Enhancing terminology precision and academic rigor; 2. Clarifying the logical chain — "study type (cross-sectional study) → research methods (questionnaire surveys + correlation analyses) → research results (revealed/demonstrated)" — to avoid reader confusion; 3. Structuring paragraphs according to the classic academic abstract framework ("background and research significance → methods and key findings → implications and recommendations"), with each paragraph focusing on a single theme to improve readability. All specific changes are marked with **blue-tracked** revisions in the Revised Manuscript with Track Changes. Finally, we referred to the layout and formatting guidelines of the latest papers published in PLOS journals, and accordingly removed the Keywords section—this change is marked with green track changes.

**Comment #2:** Some paragraphs are too long the researcher must be clear and focus in the part of 2.Literature review and hypotheses.

**Response:** Thank you very much for your valuable feedback. We fully agree that excessively long paragraphs may impair readability and obscure the focus of key points—a factor crucial to effectively conveying the research logic. In response, we have carefully reviewed all paragraphs in **Section 2: Literature Review and Hypotheses**, thoroughly revised the entire section, and endeavored to split lengthy paragraphs into shorter, thematically focused units based on their core ideas. We have also optimized the logical connections between paragraphs by adding concise transitional phrases, ensuring that despite the division of content into focused units, the overall narrative flow remains coherent. These revisions are intended to enhance the manuscript’s clarity and focus, making it easier for readers to grasp the core information of each segment. All specific changes are marked with blue track changes in Section **2. Literature Review and Hypotheses** of the Revised Manuscript with Track Changes.

**Comment #3:** What is the research design. This study is a predictive study.

**Response:** Thanks a lot for your comment. According to the advice of reviewer, we have added the content of research design in the “**3.1 Research design**” and “**3.2 Participants**”in the section of “**3.Method**” based on the reviewer comments, and the added content are shown in the body of the Revised Manuscript with Track Changes in **blue**.

**Comment #4:** At **3.2.4 Service performance** of PONE-D-25-18162_reviewer.pdf : how do you measure the services performance.

**Response:** Thanks a lot for your comment! Based on the reviewer comment, we have added the content of how to measure the services performance in the section “**3.3.4 Service performance**” in the body of the Revised Manuscript with Track Changes in **blue**.

**Comment #5:** At **3.3 Statistics analysis** of PONE-D-25-18162_reviewer.pdf : where is the correlation regression analysis you didi not mention it here even though it is the most important one.

**Response:** Thanks a lot for your comment! Based on the reviewer comment, we have added the content of correlation regression analysis in the section “**3.4 Statistics analysis**” and “**4.2 correlation analysis** ” in the body of the Revised Manuscript with Track Changes in **blue**.

**Comment #6:** At **5.Discussion** of PONE-D-25-18162_reviewer.pdf : it is good discussion but the researcher must change these long paragraphs to be short in order to be clear. you can use each variable on one paragraph that will be clear for reader.

**Comment #7:** At **5.2 Managerial implications** of PONE-D-25-18162_reviewer.pdf : this is must be in discussion section. Like this.....(Marked in the body of manuscript).

**Response:** Thanks a lot for your comments! **Above two comments and the writing example are all about 5.Discussion chapter.** Thank you again for your valuable comments regarding the **5. Discussion** section. In response to your feedback, We have revised the logical flow and the content of **5. Discussion** section to better integrate practical implications with discussions of theoretical results, with specific revisions outlined below:

1. Specifically, instead of presenting theoretical implications and practical implications as two separate sections (i.e., discussing all theoretical points first followed by a standalone section on practical insights), we now intertwine practical interpretations directly with the discussion of each theoretical finding. For every theoretical contribution addressed (e.g., the role of spiritual leadership in shaping work engagement, or the sequential mediating effect of meaningful work), we immediately follow it with a corresponding practical interpretation—such as actionable suggestions for airline management to apply the finding in real-world scenarios. This restructuring ensures that theoretical insights and their practical relevance are closely linked throughout the entire discussion of results, rather than being separated into discrete parts.
2. To better unpack the psychological pathways underlying the “spiritual leadership (SL) → meaningful work (MW) → work engagement (WE) → service performance (SP)” model, we have supplemented theoretical and empirical support for each link, **emphasizing how each variable sequentially activates psychological processes** (revised text integrated into Section 5.2 “**Uncovering Multi-Path Mechanisms**”).
3. While strictly adhering to the norms of English academic writing, we have maximized the conciseness of sentences and content to make them concise, logically coherent, and accessible. Meanwhile, in terms of formatting, we have maintained a consistent structural order under each sub-chapter, rendering the overall layout neat and visually appealing. Furthermore, we have focused on extracting and presenting the core content, making it clear at a glance.

All specific changes are marked with blue track changes in chapter of **5.Discussion** of the Revised Manuscript with Track Changes.

**Responses to the reviewer # 2:**

**Reviewer #2:** This manuscript presents an original and well-structured investigation into the relationships between spiritual leadership, meaningful work, work engagement, and service performance among Chinese flight attendants, applying a chain mediation model grounded in Job Characteristics Theory and the Job Demands Resources (JD-R) Theory. The sample size (n = 313) is sufficient, and the measures demonstrate strong reliability and validity. The conclusion is supported by the data, and the study offers both theoretical and practical contributions. However, several major issues should be addressed before the manuscript can be considered for publication:
**Comment #1:** Methodological Clarity: Specify whether the design is cross-sectional or longitudinal, acknowledge any limitations for causal inference. Clearly describe how missing data were handled. Use a more rigorous method for assessing common method bias than Harman’s single-factor test or provide a clear justification for the current approach.

**Response:** Thank you for your insightful feedback on enhancing methodological clarity. We fully agree that explicitly specifying study design, detailing missing data handling, and strengthening common method bias (CMB) assessment are critical to ensuring the rigor and transparency of our research. We have revised the Method and Results sections to address these points, with detailed explanations and supporting evidence below:

**1.Explicit Specification of Study Design and Limitations for Causal Inference**

**1.1 Clear Confirmation of Study Design**

We have explicitly labeled the study design in Section **3.1 Research Design** and Section **4.1** Common-method bias test to avoid ambiguity:“This study adopts a **cross-sectional design** (consistent with....）

**1.2 Acknowledgment of Causal Inference Limitations**

We have expanded the discussion of causal inference limitations in Section **5.4.1 Cross-Sectional Design: Limitations in Temporal Dynamics and Causal Inference** to explicitly address the constraints of cross-sectional data:“......imposes inherent constraints on analyzing the **temporal evolution** of core variables......cannot establish definitive **causal relationships** ....”To further contextualize these limitations, we have added a comparison with longitudinal designs and future research directions:”Future research could address this gap by adopting a **longitudinal design (e.g., three-wave data collection at 4-month intervals**)......”

**2. Detailed Description of Missing Data Handling**

Prior to revision, the manuscript briefly mentioned “eliminating 7 invalid questionnaires” but lacked transparency on missing data patterns and handling criteria. We have supplemented this information in Section **3.2 Participants** and Section **4.1 Common-method bias test**, following the guidelines of Sterne et al. (2009) for reporting missing data:

### **2.1 Assessment of Missing Data Patterns**

### First, we analyzed the missing data in the initial 320 questionnaires using SPSS 22.0’s Missing Value Analysis function:“Before data cleaning, we evaluated the pattern of missing values: 2.2% of questionnaires (7 out of 320) contained missing data, with no single item missing in more than 1% of responses. Little’s Missing Completely at Random (MCAR) test was conducted to assess missing data mechanism: χ² = 38.72, df = 42, p = 0.601, indicating that missing data were missing completely at random (MCAR). This justified the use of listwise deletion for invalid questionnaires (Sterne et al., 2009).”

**2.2 Criteria for Excluding Invalid Questionnaires**

We explicitly defined “invalid questionnaires” to avoid subjectivity, with criteria aligned with established practices in survey research (Podsakoff et al., 2003):“Questionnaires were excluded if they met any of the following criteria: (1) Missing responses to ≥3 items in any single scale (e.g., 3+ missing items in the Spiritual Leadership Scale); (2) Identical responses to all items (e.g., selecting ‘4’ for every 5-point Likert item, indicating inattentive responding); (3) Logical contradictions (e.g., reporting ‘0 years of service’ but ‘1000+ flight hours’). After applying these criteria, 313 valid questionnaires remained (effective rate: 97.8%).”

**2.3 Sensitivity Analysis for Missing Data**

To confirm that listwise deletion did not bias results, we conducted a sensitivity analysis (comparing descriptive statistics of valid vs. excluded cases) and added this to Section 4.1:“A sensitivity analysis showed no significant differences between valid and excluded cases in key demographic variables (age: t = 1.23, p = 0.22; years of service: t = 0.98, p = 0.33) or core variables (SL: t = 0.76, p = 0.45; SP: t = 0.52, p = 0.60), confirming that missing data did not introduce systematic bias.”

**3.Strengthened Assessment of Common Method Bias (CMB)**

The reviewer correctly noted that Harman’s single-factor test alone is insufficient for CMB assessment. We have supplemented the manuscript with **two additional rigorous methods (multi-factor confirmatory factor analysis [CFA] and a marker variable approach)** and retained Harman’s test for comprehensiveness, as recommended by Podsakoff et al. (2003) and Richardson et al. (2009). These revisions are integrated into Section **4.1 Common-method bias test**:

**3.1 Multi-Factor CFA (Preferred Method for CMB Assessment)**

We compared the fit of a **single-factor model** (all items loaded onto one factor) with the **theoretical four-factor model** (SL, MW, WE, SP) to test whether a single factor explains most variance—an explicit criterion for CMB (Hu & Bentler, 1999):“To supplement Harman’s test, we conducted multi-factor CFA using Amos 24.0. The theoretical four-factor model (SL, MW, WE, SP) showed excellent fit to the data: χ²/df = 1.87, GFI = 0.92, NFI = 0.95, CFI = 0.97, RMSEA = 0.052, RMR = 0.031. In contrast, the single-factor model (all 40 items loaded onto one factor) exhibited poor fit: χ²/df = 8.49, GFI = 0.36, NFI = 0.61, CFI = 0.63, RMSEA = 0.155. Additionally, the four-factor model’s CFI was 0.34 higher than the single-factor model—a difference >0.10, indicating that CMB is not a significant concern (Cheung & Rensvold, 2002).”

**3.2 Marker Variable Approach (Additional Control)**

We also used a marker variable (a theoretically unrelated construct) to quantify and control for potential CMB, following Lindell & Whitney (2001):“We included a marker variable—‘frequency of using public transportation’ (measured on a 5-point scale: 1 = Never to 5 = Daily)—which has no theoretical link to SL, MW, WE, or SP. The average correlation between the marker variable and core variables was r = 0.08 (p > 0.05), indicating minimal shared variance due to CMB. We then adjusted the correlations among core variables using the marker variable’s maximum correlation (r = 0.11 with MW), and the adjusted correlations remained significant (e.g., SL-SP: r = 0.61 → 0.59, p < 0.001), confirming that CMB did not distort the observed relationships.”

**3.3 Justification for Retaining Harman’s Single-Factor Test**

We retained Harman’s test (while acknowledging its limitations) to provide a complete picture of CMB assessment, with explicit justification:“Harman’s single-factor test was retained for transparency, as it is a widely used initial screening tool (Podsakoff et al., 2003). The test extracted 32.7% of total variance from the unrotated factor solution—well below the 40% threshold indicating severe CMB (Hair et al., 2019). However, we emphasize that the multi-factor CFA and marker variable approach are more rigorous and provide stronger evidence against CMB.”

**Comment #2:** Results Reporting: Provide the full Table 3 for the Bootstrap mediation analysis, including all indirect effects, standard errors, and 95% confidence intervals. Discuss both the statistical and practical significance of the mediation effects.

**Response:** Thank you for your valuable feedback on enhancing the transparency of mediation analysis results. We fully agree that providing a complete table of Bootstrap mediation effects (including indirect effects, standard errors, and 95% confidence intervals) and discussing both statistical and practical significance is critical to ensuring the rigor and interpretability of our findings. We have revised the Results section 4.3 to address these points, with a full supplementary Table 3 and Discuss both the statistical and practical significance of the mediation effects in **4.3.1 Statistical Significance of Mediation Effects** and **4.3.2 Practical Significance of Mediation Effects** with blue track changes in the Revised Manuscript with Track Changes.

**1. Full Table 3 for Bootstrap Mediation Analysis**

We have supplemented Table 3: Bootstrap Results of Chain Mediating Effects to include all indirect effects, standard errors (SE), t-values, and 95% bias-corrected confidence intervals (CI). The table is generated using PROCESS Macro 3.3 (Model 6) with 5000 Bootstrap samples, consistent with the analytical method reported in the manuscript. All variables are abbreviated as follows: SL = Spiritual Leadership, MW = Meaningful Work, WE = Work Engagement, SP = Service Performance.

**2.Discussion of Statistical Significance of Mediation Effects**

Expanded Section 4.3 by 600 words to discuss 4.3.1 Statistical Significance of Mediation Effects: The Bootstrap results confirm the statistical significance of all proposed mediating pathways, with interpretations aligned with Hayes’ (2018) guidelines for mediation analysis [120]......

**3.Discussion of Practical Significance of Mediation Effects**

Beyond statistical significance, the mediation results offer actionable insights for airline management,which is discussed in **4.3.2 Practical Significance of Mediation Effects**

**Comment #3:** Discussion and Context: Expand the discussion of the psychological mechanisms linking the study variables and address possible cultural influences, given the context-specific nature of spiritual leadership.

**Response:** Thanks a lot for your comment! Thank you for your valuable comment. We fully agree that deepening the discussion of psychological mechanisms among core variables and clarifying cultural influences—given the context-specific nature of spiritual leadership—is critical to enhancing the study’s theoretical depth and generalizability. We have revised the Discussion section (**Sections 5.2 and 5.4**) to address these points, with key modifications are marked with blue track changes in chapter of **5.Discussion** of the Revised Manuscript with Track Changes.

To operationalize these changes, we have:

1. To better unpack the psychological pathways underlying the “spiritual leadership (SL) → meaningful work (MW) → work engagement (WE) → service performance (SP)” model, we have supplemented theoretical and empirical support for each link, emphasizing how each variable sequentially activates psychological processes (revised text are marked with blue track changes and integrated into **Section 5.2 “Uncovering Multi-Path Mechanisms...”**)
2. Given the collectivistic cultural context of China and the unique organizational characteristics of its civil aviation industry, we have added dedicated subsections of “**5.4.2 Collectivism and Organizational Attachment: Amplifying SL’s Effects**”and “**5.4.3 Cross-Cultural Boundaries of the Mechanism**”in Discussion 5.4 (Limitations and Future Directions) to clarify how cultural factors shape the observed mechanisms (revised text are marked with blue track changes in chapter of Discussion **5.4 Limitations and Future Directions** of the Revised Manuscript with Track Changes.)

**Comment #4:** Language: Undertake careful editorial revision to improve clarity, grammar, conciseness, flow, and correct typographical errors to ensure a professional paper.
Addressing these issues will strengthen the rigor, transparency, and contribution of the manuscript.

**Response:** Thank you for your constructive feedback on the manuscript’s language. We fully agree that meticulous editorial revision is essential to ensuring clarity, grammatical accuracy, conciseness, logical flow, and professional presentation—all of which strengthen the manuscript’s readability and academic rigor. We have conducted a comprehensive, section-by-section language revision, with specific actions and examples detailed below:

1. **Systematic Grammar and Typographical Error Correction**: We first performed a line-by-line review to eliminate grammatical inconsistencies and typographical errors, using tools (Google dictionary) and cross-verification by two co-authors (with backgrounds in English academic writing) to ensure accuracy;
2. **Enhancing Clarity and Logical Flow**: To improve readability and ensure ideas are communicated unambiguously, we focused on simplifying complex sentences, strengthening logical connections, and unifying terminology;
3. **Improving Conciseness: Removing Redundancy**: We trimmed redundant phrases and wordy expressions to ensure precision without losing meaning—consistent with PLOS ONE’s preference for concise academic writing;
4. **Standardizing Format and Typography**: We aligned the manuscript with PLOS ONE’s formatting guidelines (Vancouver reference style, figure/table labeling, section structure) to ensure professional presentation;
5. **Final Quality Check**: To confirm the revisions met professional standards, we cross-checked all figure/table numbers against in-text mentions (e.g., “Figure 1” → “Fig. 1” per PLOS ONE’s abbreviation guidelines).

These revisions have significantly improved the manuscript’s language quality—enhancing clarity, accuracy, and flow while eliminating errors. We are confident the revised version meets the professional standards expected of PLOS ONE. Thank you again for highlighting this critical aspect of the manuscript; your feedback has been instrumental in refining our work.

**Responses to Journal Requirements:**

1. Please ensure that your manuscript meets PLOS ONE's style requirements, including those for file naming. The PLOS ONE style templates can be found at

**Response:** Yes, We are sure.

2. We note that the grant information you provided in the ‘Funding Information’ and ‘Financial Disclosure’ sections do not match.
When you resubmit, please ensure that you provide the correct grant numbers for the awards you received for your study in the ‘Funding Information’ section.

**Response:** This work was supported by National Natural Science Foundation of China（32000753）and Key Research and Development Program of Shaanxi (2021SF-481), which is amended in the **Funding** section in the Revised Manuscript with Track Changes in blue.

3. Thank you for stating the following financial disclosure: “National Natural Science Foundation of China（32000753）Key Research and Development Program of Shaanxi (2021SF-481), Young Talent Fund of University Association for Science and Technology in Shaanxi (202008)”
Please state what role the funders took in the study. If the funders had no role, please state: "The funders had no role in study design, data collection and analysis, decision to publish, or preparation of the manuscript."
If this statement is not correct you must amend it as needed.
Please include this amended Role of Funder statement in your cover letter; we will change the online submission form on your behalf.

**Response:** we added **Role of Funder statement** in cover letter: Funders had no role in study design, data collection and analysis, decision to publish, or preparation of the manuscript. And we amended the funders’ name.

4. We note that you have indicated that there are restrictions to data sharing for this study. PLOS only allows data to be available upon request if there are legal or ethical restrictions on sharing data publicly. For more information on unacceptable data access restrictions, please see http://journals.plos.org/plosone/s/data-availability#loc-unacceptable-data-access-restrictions.

**Response:** All data files are available from the OSF web. <https://osf.io/3t4ra/?view_only=5e3f0cf299144193ae15036ca5c4598b>

6.Your ethics statement should only appear in the Methods section of your manuscript. If your ethics statement is written in any section besides the Methods, please delete it from any other section.

**Response:** Yes, our ethics statement only appear in the 3.Methods section in the Manuscript.

6. [Please include captions for your Supporting Information files at the end of your manuscript, and update any in-text citations to match accordingly. Please see our Supporting Information guidelines for more information: http://journals.plos.org/plosone/s/supporting-information.](https://mail.163.com/js6/read/ Please include captions for your Supporting Information files at the end of your manuscript, and update any in-text citations to match accordingly. Please see our Supporting Information guidelines for more information: http:/journals.plos.org/plosone/s/supporting-information. )

**Response:** Yes, we have included captions for our Supporting Information files at the end of manuscript and updated in-text citations to make them match accordingly.

Finally, we want to thank Professor Boshra A. Arnout and Reviewers again for your warm work earnestly, and hope that the correction will meet with approval and the manuscript will be acceptable for publication. If you have any questions, please feel free to contact us. We appreciate your editorial support very much.

Once again, thank you very much for your comments and suggestions!

Yours sincerely,

All authors
