# Supplementary material for: Spiritual leadership and service performance among Chinese flight attendants: The mediating effects of meaningful work and work engagement
Source: PLoS One. 2026 May 21;21(5):e0324405. doi: 10.1371/journal.pone.0324405 (PMC13193339; doi:10.1371/journal.pone.0324405)
Supplement: Attachment — Submitted filename: Response_to_reviewers_auresp_2.docx [file pone.0324405.s004.docx]

Dear Professor Boshra A. Arnout and Reviewers,

Thank you for your letter and for the Reviewers’ comments concerning our manuscript entitled “Spiritual leadership and service performance among Chinese flight attendants: The mediating effects of meaningful work and work engagement” (ID: PONE-D-25-18162). Those comments are all valuable and very helpful for revising and improving our paper, as well as the important guiding significance to our research. We have studied the comments carefully and have made correction to meet with approval. The revised portions of the content are marked in the file of Revised Manuscript with Track Changes. Also, we have modified the wording mistakes in the entire manuscript. **Since Reviewer #1 has not provided any negative feedback, only the issues where Reviewer #2's comments are marked as "no" in the comments list are displayed below**.The main corrections in the paper and the detailed point-by-point responds to the reviewer’s comments are as follow:

**List of Responses**

**Responses to the reviewer # 2:**

**4.Have the authors made all data underlying the findings in their manuscript fully available?**
The [PLOS Data policy](http://www.plosone.org/static/policies.action#sharing) requires authors to make all data underlying the findings described in their manuscript fully available without restriction, with rare exception (please refer to the Data Availability Statement in the manuscript PDF file). The data should be provided as part of the manuscript or its supporting information, or deposited to a public repository. For example, in addition to summary statistics, the data points behind means, medians and variance measures should be available. If there are restrictions on publicly sharing data—e.g. participant privacy or use of data from a third party—those must be specified.

**Reviewer #2:** No.

**Comment:** Thank you for your valuable feedback on data transparency. We fully agree that making the underlying data points (beyond summary statistics) publicly available is critical for reproducibility, and we have addressed this requirement comprehensively:

### 1. Public Availability of Full Data Points

All raw and processed data supporting the summary statistics (means, medians, variances, correlations, and regression/mediation results) ,which stored in excel file, have been uploaded to the Open Science Framework (OSF) with no access restrictions. The publicly shared dataset includes:

- Individual-level raw responses to all questionnaire items (de-identified to protect privacy);
- Cleaned data files (with invalid cases excluded, as detailed in Section 3.2) containing each participant’s scores on spiritual leadership (SL), meaningful work (MW), work engagement (WE), service performance (SP), and control variables (age, years of service, flight time, position);

The permanent OSF link is: [https://osf.io/3t4ra/?view_only=5e3f0cf299144193ae15036ca5c4598b](https://osf.io/3t4ra/?view_only=5e3f0cf299144193ae15036ca5c4598b)

### 2. No Restrictions on Data Sharing

There are no legal, ethical, or third-party restrictions on sharing the data:

- Participant privacy is protected via full de-identification (no names, employee IDs, or other personally identifiable information);
- The dataset was collected through a collaborative research project with Tibet Airlines, and the airline has provided written authorization for public data sharing (a copy of the authorization is available upon request to the editorial office);
- No commercial or proprietary information is included in the shared data—all variables are derived from standardized psychological scales or demographic self-reports.

### 3. Updated Manuscript Statement

We have revised the “Availability of data and materials” section in the manuscript to explicitly, marked in blue, confirm the inclusion of underlying data points:

“All data points supporting the summary statistics (means, medians, variances, and inferential analyses) are available from the Open Science Framework (OSF) at [https://osf.io/3t4ra/?view_only=5e3f0cf299144193ae15036ca5c4598b](https://osf.io/3t4ra/?view_only=5e3f0cf299144193ae15036ca5c4598b). The dataset includes de-identified raw responses, cleaned data files, and variable coding schemes, with no restrictions on access or use.”

These revisions ensure full compliance with PLOS ONE’s data transparency standards, allowing other researchers to verify our statistical results and conduct secondary analyses. Thank you again for highlighting this critical aspect of research reproducibility.

**6. Review Comments to the Author**

Please use the space provided to explain your answers to the questions above. You may also include additional comments for the author, including concerns about dual publication, research ethics, or publication ethics. (Please upload your review as an attachment if it exceeds 20,000 characters)

**Reviewer #2:** The authors have substantially strengthened the manuscript in response to the previous review. The revised version demonstrates clear improvements in theoretical articulation, methodological transparency, and presentation quality. The conceptual model linking spiritual leadership, meaningful work, work engagement, and service performance is well grounded in the Job Characteristics Theory and the Job Demands Resources (JDR) framework, and the findings are consistent with the proposed mediation pathways.

The statistical analyses are appropriate and competently executed, and the reported measures exhibit sound reliability and validity. The discussion now provides a stronger theoretical and practical interpretation of the results, particularly regarding the motivational mechanisms underlying the observed relationships.

**Only** **a few minor issues** **remain before publication.** Specifically, the authors should **(1) explicitly clarify the study design and acknowledge its cross-sectional limitation for causal inference, (2) describe how missing data were handled, (3) provide a more detailed explanation of the procedure used to assess common method bias, and (4) ensure full compliance with PLOS ONE’s open data policy by making the dataset and analysis syntax publicly available.**

Overall, this is a well executed and meaningful study that makes a valuable contribution to the literature on spiritual leadership and employee engagement. Subject to these minor revisions, the manuscript is suitable for publication.

**Comments :** Thank you for your positive evaluation of our revised manuscript and your constructive feedback on the remaining minor issues. We greatly appreciate your recognition of the improvements in theoretical articulation, methodological transparency, and presentation quality. We have thoroughly addressed each of your specific suggestions to further enhance the manuscript’s rigor, transparency, and compliance with PLOS ONE’s standards, with detailed responses below:

**(1) Explicitly clarify the study design and acknowledge its cross-sectional limitation for causal inference**

We have explicitly clarified the study design and thoroughly acknowledged its limitations for causal inference **in Section 3.1 Research Design and Section 5.4.1 Cross-Sectional Design: Limitations in Temporal Dynamics and Causal Inference**, with targeted revisions as follows:

**Step1: Clear specification of study design in Section 3.1 Research Design**:“This study adopts a cross-sectional design, which involves collecting data from flight attendants via a single-time-point psychological questionnaire survey. Consistent with prior aviation research on leadership and service performance [7, 20], this design is specifically tailored to our core research objective—exploring the correlational relationships and mediating mechanisms among spiritual leadership (SL), meaningful work (MW), work engagement (WE), and service performance (SP)—rather than verifying temporal causality between variables. The cross-sectional approach effectively addresses the logistical challenges of accessing flight attendants (a population with irregular work schedules) and allows for initial validation of the proposed theoretical model.”

**Step2: Explicit acknowledgment of causal inference limitations in 5.4.1 Cross-Sectional Design: Limitations in Temporal Dynamics and Causal Inference**:“

This study adopted a cross-sectional design [120, 136], which imposes inherent constraints on analyzing the temporal evolution of core variables:

- It precludes the examination of how flight attendants’ psychological states (e.g., fluctuations in meaningful work, work engagement) and perceptions of spiritual leadership change over time (e.g., before/after leadership training, during peak/off-peak flight seasons).Consequently, the predictive validity of the observed correlations is limited—we cannot establish definitive causal relationships between spiritual leadership, meaningful work, work engagement, and service performance, only associations.
- Future research could address this gap by adopting a longitudinal design (e.g., three-wave data collection at 4-month intervals) to track the temporal dynamics of these variables. This approach would allow for stronger causal inference via cross-lagged panel analysis, as recommended by [145] for testing mediation models in organizational psychology.”

These revisions explicitly distinguish the study’s design purpose from its limitations, provide concrete examples of unresolved causal ambiguities, and offer actionable directions for future research—aligning with PLOS ONE’s emphasis on methodological transparency.

(2) **Describe how missing data were handled**

Prior to revision, the manuscript briefly mentioned “eliminating 7 invalid questionnaires” but lacked transparency on missing data patterns and handling criteria. We have supplemented this information in Section **3.2 Participants** and Section **4.1 Common-method bias test**, following the guidelines of Sterne et al. (2009) for reporting missing data:

- **Assessment of Missing Data Patterns**

First, we analyzed the missing data in the initial 320 questionnaires using SPSS 22.0’s Missing Value Analysis function:“Before data cleaning, we evaluated the pattern of missing values: 2.2% of questionnaires (7 out of 320) contained missing data, with no single item missing in more than 1% of responses. Little’s Missing Completely at Random (MCAR) test was conducted to assess missing data mechanism: χ² = 38.72, df = 42, p = 0.601, indicating that missing data were missing completely at random (MCAR). This justified the use of listwise deletion for invalid questionnaires (Sterne et al., 2009).”

- **Criteria for Excluding Invalid Questionnaires**

We explicitly defined “invalid questionnaires” to avoid subjectivity, with criteria aligned with established practices in survey research (Podsakoff et al., 2003):“Questionnaires were excluded if they met any of the following criteria: (1) Missing responses to ≥3 items in any single scale (e.g., 3+ missing items in the Spiritual Leadership Scale); (2) Identical responses to all items (e.g., selecting ‘4’ for every 5-point Likert item, indicating inattentive responding); (3) Logical contradictions (e.g., reporting ‘0 years of service’ but ‘1000+ flight hours’). After applying these criteria, 313 valid questionnaires remained (effective rate: 97.8%).”

- **Sensitivity Analysis for Missing Data**

To confirm that listwise deletion did not bias results, we conducted a sensitivity analysis (comparing descriptive statistics of valid vs. excluded cases) and added this to Section 4.1:“A sensitivity analysis showed no significant differences between valid and excluded cases in key demographic variables (age: t = 1.23, p = 0.22; years of service: t = 0.98, p = 0.33) or core variables (SL: t = 0.76, p = 0.45; SP: t = 0.52, p = 0.60), confirming that missing data did not introduce systematic bias.”

(3) **Provide a more detailed explanation of the procedure used to assess common method bias (CMB)**

We have expanded the CMB assessment description in **Section 4.1 Common-method Bias Test** to clarify the “procedural control + post-hoc statistical validation” dual approach, with step-by-step details:

**Step 1: Procedural controls during data collection (to mitigate CMB ex ante)**

- Established a strategic partnership with Tibet Airlines to standardize sampling and ensure data quality;
- Randomly sampled participants from the airline’s official roster to avoid selection bias;
- Provided detailed information sheets (on study purpose, data usage) and clear response instructions (e.g., “avoid uniform responses” with examples) to minimize ambiguity;
- Guaranteed anonymity, confidentiality, and no adverse career impacts, with voluntary participation—reducing response bias (e.g., social desirability).

**Step 2: Post-hoc statistical validation (three complementary methods)**

**1.Harman’s single-factor test** (initial screening): “This test—retained for transparency as a widely used initial screening tool [122]—extracted 32.7% of total variance from the unrotated factor solution, well below the 40% threshold indicating severe CMB [149].”

**2.Multi-factor confirmatory factor analysis** (CFA) (preferred method): “To supplement Harman’s test, we performed multi-factor CFA in Amos 24.0. The theoretical four-factor model (spiritual leadership [SL], meaningful work [MW], work engagement [WE], service performance [SP]) exhibited excellent data fit: χ²/df = 1.87, goodness-of-fit index (GFI) = 0.92, normed fit index (NFI) = 0.95, comparative fit index (CFI) = 0.97, root mean square error of approximation (RMSEA) = 0.052, and root mean square residual (RMR) = 0.031. By contrast, the single-factor model (all 40 items loading onto one factor) showed poor fit: χ²/df = 8.49, GFI = 0.36, NFI = 0.61, CFI = 0.63, RMSEA = 0.155. Additionally, the four-factor model’s CFI was 0.34 higher than the single-factor model—a difference > 0.10, indicating CMB is not a significant concern [148].”

**3.Marker variable approach** (additional control): “We further employed a marker variable (a theoretically unrelated construct) to quantify and control for potential CMB, following Lindell & Whitney [150]. The marker variable—“frequency of using public transportation”—was assessed on a 5-point Likert scale (1 = Never, 5 = Daily) and has no theoretical link to SL, MW, WE, or SP. The average correlation between the marker variable and core variables was r = 0.08 (p > 0.05), indicating minimal shared variance attributable to CMB. We then adjusted correlations among core variables using the marker variable’s maximum correlation (r = 0.11 with MW); adjusted correlations remained significant (e.g., SL-SP: r = 0.61 → 0.59, p < 0.001), confirming CMB did not distort observed relationships between core variables.”

**(4) Ensure full compliance with PLOS ONE’s open data policy**

### 1. Public Availability of **Dataset and Analysis Syntax**

All raw and processed **Dataset and Analysis Syntax** supporting the summary statistics (means, medians, variances, correlations, and regression/mediation results) have been uploaded to the Open Science Framework (OSF) with no access restrictions. The publicly shared dataset includes:

- Individual-level raw responses to all questionnaire items (de-identified to protect privacy);
- Cleaned data files (with invalid cases excluded, as detailed in Section 3.2) containing each participant’s scores on spiritual leadership (SL), meaningful work (MW), work engagement (WE), service performance (SP), and control variables (age, years of service, flight time, position);
- **Analysis Syntax**

The permanent OSF link is: [https://osf.io/3t4ra/?view_only=5e3f0cf299144193ae15036ca5c4598b](https://osf.io/3t4ra/?view_only=5e3f0cf299144193ae15036ca5c4598b)

### 2. No Restrictions on Data Sharing

There are no legal, ethical, or third-party restrictions on sharing the data:

- Participant privacy is protected via full de-identification (no names, employee IDs, or other personally identifiable information);
- The dataset was collected through a collaborative research project with Tibet Airlines, and the airline has provided written authorization for public data sharing (a copy of the authorization is available upon request to the editorial office);
- No commercial or proprietary information is included in the shared data—all variables are derived from standardized psychological scales or demographic self-reports.

All four minor issues you raised have been addressed with targeted, transparent revisions. We have enhanced the manuscript’s methodological rigor, clarity, and compliance with PLOS ONE’s standards while preserving its core contributions. We sincerely thank you for your insightful feedback, which has been instrumental in refining our work.
