# Supplementary material for: Spiritual leadership and service performance among Chinese flight attendants: The mediating effects of meaningful work and work engagement
Source: PLoS One. 2026 May 21;21(5):e0324405. doi: 10.1371/journal.pone.0324405 (PMC13193339; doi:10.1371/journal.pone.0324405)
Supplement: Attachment — Submitted filename: Response_to_reviewers_auresp_3.docx [file pone.0324405.s005.docx]

Dear Professor Boshra A. Arnout and Dr. Abdallah,

Thank you for your insightful and constructive comments on our manuscript entitled “Spiritual leadership and service performance among Chinese flight attendants: The mediating effects of meaningful work and work engagement” (ID: PONE-D-25-18162). We greatly appreciate the editorial team’s guidance and Reviewer #3’s rigorous evaluation. **Since our prior comments have been fully addressed, we focus below solely on the items marked “No” in Reviewer #3’s comments list.** Your feedback on strengthening the manuscript’s rigor, reproducibility, and evidentiary support is invaluable, and we have **carefully addressed all major and minor issues with targeted revisions**, enhanced reporting, and strict adherence to PLOS ONE’s academic and data policies. All revised content is clearly marked with Track Changes in the revised manuscript, and supporting materials are supplemented as required. Below is our detailed point-by-point response to these comments, along with the corresponding revisions made to the manuscript.

**List of Responses**

**Responses to the reviewer # 3:**

Reviewer #3: Decision: Major Revision — key elements needed to judge rigor, reproducibility, and evidentiary support need strengthening.

**Statistical analysis: Partly — the analytical approach may be reasonable, but reporting is not yet sufficient to assess robustness (e.g., model justification, assumption checks, precision, and sensitivity checks).**

**Response:** We thank the reviewer for this insightful comment, which highlights the importance of transparent and rigorous statistical reporting. We have carefully revised the relevant sections of the manuscript to address this critical concern: we now provide explicit justifications for our analytical model choices, detailed results of key assumption testing (e.g., normality, multicollinearity), and comprehensive reporting of effect sizes alongside their precision estimates (e.g., 95% confidence intervals) in the Section 3.4 Statistic analysis. These additions ensure that our analytical approach is fully justified, reproducible, and statistically robust, thereby strengthening the internal and external reliability of our findings. Specific, point-by-point revisions in response to this comment are outlined below.

**Data availability: No (if applicable) — the manuscript does not provide the minimal dataset (or a working repository link) required to replicate findings under PLOS data policy, except in rare, justified cases.**

**Response:** Thank you for your feedback on data transparency—we fully agree that comprehensive access to underlying data is critical for research reproducibility, and we have enhanced our documentation to provide greater clarity on the structure, content, and accessibility of the shared dataset (with explicit details on Excel and SPSS files as requested):

**1. Comprehensive Public Availability of All Underlying Data Files**

All raw, cleaned, and processed data required to replicate our findings (including data points behind summary statistics and inferential analyses) have been permanently deposited in the **Open Science Framework (OSF)** with unrestricted view-only access for all researchers. The repository includes structured files with clear categorization, as detailed below:

**(1) Excel File: “datasets and processed information.xlsx” (Core Dataset)**

This file contains four dedicated sheets to facilitate easy navigation and replication, with explicit sample size labeling:

- Sheet 1: raw_data (N = 320)

Individual-level raw responses to every questionnaire item (e.g., all items of the Spiritual Leadership Scale, Work as Meaning Inventory) from the initial 320 participants, before data cleaning. All entries are fully de-identified to protect participant privacy.

- Sheet 2: cleaned_data (N = 313)

Refined dataset after excluding 7 invalid cases per our pre-specified criteria in Section 3.2 (e.g., missing ≥3 items in a single scale, logical contradictions). It includes participant-specific scores for:

Core variables: spiritual leadership (SL), meaningful work (MW), work engagement (WE), service performance (SP);

Control variables: age, years of service, total flight time, job position, gender, highest education level and marital status.

- Sheet 3: processed_data

Aggregated and derived data supporting all statistical results reported in the manuscript, including:

Summary statistics: means, medians, and variances for all core and control variables;

Inferential analysis outputs: correlation matrices, regression coefficients, and mediation effect results (including indirect effects, standard errors, and 95% confidence intervals).

**(2) SPSS Data File: “313 sample.sav”**

This dedicated .sav file provides explicit coding instructions and value labels for all categorical demographic variables, which can be directly imported and applied in SPSS. Key examples include:

- Gender: 1 = Male, 2 = Female;
- Job position: 1 = Flight Attendant Trainee, 2 = Economy Class Flight Attendant, 3 = Business/First Class Flight Attendant, 4 = Purser, 5 = Cabin Manager;
- Marital status: 1 = Single (Never Married), 2 = Married, 3 = Other Marital Status;
- Educational qualification: 1 = High school or below, 2 = College diploma (3-year tertiary education), 3 = Bachelor’s degree, 4 = Master’s degree, 5 = Doctoral degree or above.

The permanent, fully functional OSF repository link is: <https://osf.io/3t4ra/?view_only=5e3f0cf299144193ae15036ca5c4598b>. We have verified this link across multiple devices and regions to confirm global accessibility—no login, registration, or additional access restrictions are required. The Excel file can be opened with standard spreadsheet software, and the .sav file is directly compatible with SPSS (compatible with SPSS 22.0 and above), allowing researchers to replicate our variable coding and data processing steps seamlessly.

**2. No Restrictions on Data Sharing**

There are no legal, ethical, or third-party barriers to accessing or using the data:

Participant privacy is rigorously protected through full de-identification (no names, employee IDs, flight details, or other personally identifiable information);

Tibet Airlines (our research partner) has provided written authorization for public data sharing (a copy of this authorization is available to the editorial office upon request);

The dataset contains no commercial, proprietary, or sensitive information—all variables are derived from standardized psychological scales (e.g., Fry et al.’s Spiritual Leadership Scale, Schaufeli et al.’s UWES-9) or non-identifiable demographic self-reports.

**3. Updated and Explicit Manuscript Statement**

We have revised the “Availability of data and materials” section in the manuscript (**Page 40**) to clearly articulate the full scope of shared data files and correct the file format:

“*All data points supporting the descriptive statistics (means, medians, variances) and inferential analyses are available on the Open Science Framework (OSF) at* [*https://osf.io/kygps/overview?view_only=b9079cb5039a495aacb7e5005f1444ec*](https://osf.io/kygps/overview?view_only=b9079cb5039a495aacb7e5005f1444ec)*. The dataset contains de-identified raw responses, cleaned data files, processed data, and variable coding schemes, with no restrictions on access or usage.*”

These measures ensure full compliance with PLOS ONE’s data transparency standards, enabling other researchers to independently verify our statistical results, replicate the mediation models, and conduct secondary analyses with minimal barriers. We appreciate your diligence in upholding research reproducibility standards.

**Major issues for revision**

**Conceptual framing and contribution**

1. **Clarify the conceptual/theoretical lens, the specific literature gap, and the manuscript’s intended contribution.**

**Response:** We have explicitly clarified the conceptual/theoretical lens, specific literature gap, and the manuscript’s intended contribution by integrating a concise summary into the Introduction section (synthesized with the existing discussion of the study’s uniqueness and theoretical model).

The clarified content includes: (1) The study’s dual theoretical foundation (Job Characteristics Theory and Job Demands-Resources Theory) and their synergistic explanation of the sequential mechanism (SL→MW→WE→SP); (2) Three critical research gaps (lack of SL-SP empirical evidence in flight attendants, underdeveloped sequential mediation pathways, and insufficient cross-theoretical integration); (3) Corresponding theoretical, empirical, and practical contributions. The revised content is presented in Page 6, Paragraph 2: “*In general, thus far, this study is the first to investigate the aforementioned relationships simultaneously from the perspectives of organizational psychology, positive psychology, and occupational psychology, using data gathered from airline flight attendants. Grounded in Job Characteristics Theory (JCT) and Job Demands-Resources (JD-R) Theory, JCT explains how spiritual leadership (SL) enhances meaningful work (MW) via task significance alignment, while JD-R Theory illustrates SL and MW as resources fostering work engagement (WE) and service performance (SP). Three critical gaps motivate this research: (1) no empirical SL-SP link in flight attendants; (2) underdeveloped MW→WE sequential mediation; (3) lack of cross-theoretical integration. Corresponding contributions include: (1) integrating JCT-JD-R to validate the SL→MW→WE→SP model; (2) providing large-sample empirical evidence; (3) offering actionable strategies for airline management. The theoretical chain mediation model of this study is depicted in Figure 1. By doing so, this study not only advances the literature on the relationship between spiritual leadership and service performance, but also foregrounds and extends the psychological mechanism through which spiritual leadership positively impacts flight attendants’ service performance, offering practical clues for airline managers to refine their practices.*”

2. **Ensure RQs/hypotheses are explicitly motivated by prior empirical work and constructs are consistently defined/operationalized.**

**Response:** We have ensured that all research hypotheses are explicitly motivated by prior empirical work, and all core constructs are consistently defined and operationalized throughout the manuscript, with detailed elaborations and empirical supports presented in the relevant sections as follows:

- **Hypotheses motivated by prior empirical work:**

Each hypothesis is rigorously grounded in existing empirical studies and literature on spiritual leadership, meaningful work, work engagement and service performance (especially in the aviation context). The empirical supports for the hypotheses are explicitly presented in the **2. Literature review and hypotheses section (Page 7–12)**, with key prior findings cited to justify the proposed relationships (e.g., the positive effect of spiritual leadership on job performance in aviation [28], the mediating role of meaningful work between leadership and performance [36, 82], and the sequential link between meaningful work and work engagement [46, 48]).

- **Consistent definition and operationalization of constructs:**

Core constructs (SL, MW, WE, SP) are uniformly defined in the **Introduction (Page 4–6)** and **Literature review and hypotheses section (Page 7–12)** with classic/recent study references;

All constructs are systematically operationalized in the **Measures subsection (Page 13–15) of the Method section**, where valid and reliable established scales are adopted for each construct, with consistent measurement criteria (Likert scale ranges, internal consistency coefficients) reported and applied in subsequent data analysis.

3. **Update and broaden the recent peer‑reviewed literature.**

**Response:** Thank you for your comment. We confirm the manuscript has comprehensively integrated 7 peer-reviewed studies published after 2020, with explicit in-text citations that reinforce theoretical, contextual, and methodological rigor. Details are as follows:

(1) Spiritual leadership & performance:

- Ali et al. [28] (2020): Cited in **Introduction (Page 4) and Discussion (Page 31)** to support spiritual leadership’s effectiveness in high-risk industries:

Introduction: “*Spiritual leadership has been proved to work well in promoting task performance... and safety performance for airline pilots [28].*”

Discussion: “*Existing limited studies are either confined only to any one or two of the three key constructs: Spiritual Leadership (SL), Service Performance (SP), and Flight Attendants (e.g., [57, 36] on SL and job performance; Ali et al. [28] on SL and pilots’ safety behavior; Karatepe & Talebzadeh [20] on flight attendants’ service recovery performance*”

- Pio [57] (2022): Cited in **Discussion (Page 31)** to align with spiritual leadership-performance mediation: “*Existing limited studies... (e.g., [57, 36] on SL and job performance)*”.

(2) Aviation-specific service performance:

- Shen et al. [9] (2021): Cited in **Introduction (Page 3)** to emphasize service quality’s importance: “*Delivering high-quality service and retaining loyal passengers are critical for success [6, 9, 50, 51].*”
- Shin et al. [70] (2022): Cited in **Discussion (Page 31)** to confirm industry context uniqueness: “*This aligns with prior research on attendants’ WE [18] and Li et al.’s [114] finding that organizational trust predicts safety behavior via mediating variables in civil aviation, confirming the applicability of mediation models in the aviation context. It also echoes Shin et al.’s [70] focus on flight attendants’ service performance as a target of contextual and leadership factors.*”

(3) Methodological & theoretical support:

- Lai et al. [97] (2020): Cited in **Discussion (Page 36)** to validate mediation pathways: “*This aligns with prior studies documenting sequential mediation of MW and WE in other contexts [48, 112, 128]) but adds specificity by grounding the mechanism in JD-R Theory, and echoes Lai et al.’s [97] finding that transformational leadership predicts job performance via work engagement.*”
- Wang et al. [121] (2022): Cited in **Statistical Analysis (Page 20)** to support chain mediation: “…*a method recommended for mediation testing due to its robustness to non-normal data distributions [119], which is that a significant mediating effect was indicated if bootstrap confidence interval (CI) does not include zero based on 5000 random samples [120, 121].*”
- Li et al. [114] (2021): Cited in **Literature review and hypotheses (Page 13)** to support hypothesis logic: “*Meaningful work is positively related to flight attendants’ work engagement. In fact, individuals' real-life work environments are often complex and dynamic, shaped by multiple factors that influence behavior [114],*”

**Methods transparency and reproducibility**

**Response:** Thank you for your comment regarding methods transparency and reproducibility. We confirm that all requested elements—study design justification, detailed sampling procedures, comprehensive measure descriptions, standardized procedures, and ethics/confidentiality safeguards—are thoroughly addressed in the **Method section of the manuscript (Pages 15–22)**. Below is a detailed breakdown of how each requirement is fulfilled, with specific references to the corresponding sections.

4. **State and justify the study design; detail sampling (criteria, recruitment, setting, sample-size rationale, flow/attrition);**

**Response:** Thank you for your valuable comment on the study design and sampling details. We have supplemented and clarified the relevant information in the revised manuscript to ensure full transparency and rigor of the research methods.

(1) **Study design and justification**

The study adopts a cross-sectional design (Section 3.1, Page 14-15), explicitly justified by the core research objective:

“*This study adopts a cross-sectional design, which involves collecting data from flight attendants via a single-time-point psychological questionnaire survey. Consistent with prior aviation research on leadership and service performance [7, 20], this design is specifically tailored to our core research objective—exploring the correlational relationships and mediating mechanisms among spiritual leadership (SL), meaningful work (MW), work engagement (WE), and service performance (SP)—rather than verifying temporal causality between variables. The cross-sectional approach effectively addresses the logistical challenges of accessing flight attendants (a population with irregular work schedules) and allows for initial validation of the proposed theoretical model.*”

(2) **Sampling details**

We revised Section 3.2 Participants to include additional details regarding recruitment flow, research setting, and participant selection criteria, as presented below (Page 15-16, Section 3.2):

- **Criteria (Page 16, Paragraph 1):** “*After retrieving all questionnaires, the completeness and validity of the collected data were systematically assessed, with 7 invalid questionnaires excluded in accordance with pre-determined criteria [122]. The specific exclusion criteria for questionnaires were as follows: (1) Missing responses to ≥3 items in any single scale (e.g., more than 3 missing items in the Spiritual Leadership Scale); (2) Identical responses to all items (e.g., selecting ‘4’ for every 5-point Likert item, indicating inattentive responding); (3) Obvious logical contradictions in response information (e.g., reporting ‘0 years of service’ but ‘1000+ flight hours’). Regarding sample flow and attrition, the above criteria yielded a total of 313 valid questionnaires, corresponding to an effective response rate of 97.8%. Before data cleaning, the pattern of missing values was evaluated: 2.2% of the questionnaires (7 out of 320) contained missing data, with no single item having a missing rate exceeding 1%. Little’s Missing Completely at Random (MCAR) test was further conducted to assess the missing data mechanism, with the results (χ² = 38.72, df = 42, p = 0.601) indicating that the missing data were completely random (MCAR). This statistical evidence justified the use of listwise deletion for handling invalid questionnaires [146].*”
- **Recruitment & setting:** “*A total 320 in-service Chinese flight attendants were randomly selected as initial participants from Tibet Airlines via the company’s internal collaborative channels across its operational branches.*” and the details of recruitment also described in Section 3.1 Research design: “*Questionnaires were administered electronically via the professional data collection platform Wenjuanxing (https://www.wjx.cn/) and distributed through WeChat, China's most popular social networking application. With assistance from our research partners at Tibet Airlines, survey instruments and information statements were sent to randomly selected flight attendants from company personnel lists.*”
- **Sample-size rationale:**

**“***The sample size was determined based on the statistical requirements of mediation effect analysis and confirmatory factor analysis, with the initial 320 participants meeting the generally recommended minimum sample size threshold (≥200) for such analyses, thus ensuring adequate statistical test power [117,120].***”**

- **Flow/attrition:** A clear participant flow is reported: 320 initially sampled→7 excluded due to invalid data (criteria: ≥3 missing items in any scale, identical responses to all items, or logical contradictions)→313 valid participants (Section 3.2, Page 16). Additionally, Little’s Missing Completely at Random (MCAR) test (χ² = 38.72, df = 42, p = 0.601) confirmed missing data were random, justifying listwise deletion.

5. **Describe measures/data sources (validation and reliability where relevant);**

**Response:** All measures adopted in this study are established, widely used scales in organizational and occupational psychology, with clear data sources, and rigorous validation and reliability testing as reported in the manuscript:

- Spiritual Leadership (SL, see Page 17, Section 3.3.1):

“*In the present study, this scale showed excellent reliability with a Cronbach’s α coefficient of 0.973. Confirmatory factor analysis was conducted to analyze the structural validity of the constructs. The results in the present study showed a good fit with the data: χ2 /df = 2.357, GFI = 0.932, NFI = 0.972, TLI = 0.971, CFI = 0.983, RMR=0.031, RMSEA = 0.066.*”

- Meaningful Work (MW, see Page 17, Section 3.3.2):

“*In the present study, this scale showed excellent reliability with a Cronbach’s α coefficient of 0.924. Confirmatory factor analysis was conducted to analyze the structural validity of the constructs. The results in the present study showed a good fit with the data: χ2 /df = 1.612, GFI = 0.975, NFI = 0.984, TLI = 0.989, CFI = 0.994, RMR=0.020, RMSEA = 0.044.*”

- Work Engagement (WE, see Page 17, Section 3.3.3): “*In the present study, this scale showed excellent reliability with a Cronbach’s α coefficient of 0.966. Confirmatory factor analysis was conducted to analyze the structural validity of the constructs. The results in the present study showed a good fit with the data: χ2 /df = 1.677, GFI = 0.979, NFI = 0.991, TLI = 0.993, CFI = 0.996, RMR=0.022, RMSEA = 0.047.*”
- Service Performance (SP, see Page 17, Section 3.3.4): “*In the present study, this scale showed excellent reliability with a Cronbach’s α coefficient of 0.948. Confirmatory factor analysis was conducted to analyze the structural validity of the constructs. The results in the present study showed a good fit with the data:* *χ2 /df = 2.149, GFI = 0.993, NFI = 0.997, TLI = 0.995, CFI = 0.998, RMR=0.004, RMSEA = 0.061*”

6. **Specify procedures (timing, training/standardization, bias minimization);**

**Response:** Thank you for your comments. We specified the detailed procedures as follows:

- Timing: We have clarified the specific post-COVID-19 data collection period**, “***Data were collected between September–December 2021 (post-COVID-19 recovery period).***”**
- Standardization: All questionnaires were distributed and collected via the professional data collection platform Wenjuanxing (https://www.wjx.cn/), with fixed response logic (e.g., mandatory completion of core items to reduce missing data) and automatic data encoding to ensure consistency of data format (Supplemented in Section 3.1, Page 15–16):

“*Questionnaires were administered electronically via the professional data collection platform Wenjuanxing (https://www.wjx.cn/) and distributed through WeChat, China's most popular social networking application. With assistance from our research partners at Tibet Airlines, survey instruments and information statements were sent to randomly selected flight attendants from company personnel lists.*”

“*Given flight attendants' work characteristics, the electronic format enabled participants to complete self-report measures anytime and anywhere by scanning the questionnaire QR code via WeChat, thereby enhancing both response rates and data quality.*”

- Bias minimization: We confirm that comprehensive procedural and statistical bias control strategies have been fully implemented in the study, with detailed descriptions in the manuscript (Section 3.2 Participants, Page 16; Section 4.1 Common-method bias test, Page 23–25). The specific measures are as follows:

(1) Procedural bias minimization

**Random sampling**: 320 in-service flight attendants were **randomly** selected from Tibet Airlines’ official roster across its operational branches, ensuring sample representativeness of the target population (Section 3.2, Page 16).

**Anonymous survey**: Participants were explicitly assured of strict confidentiality, with no personal identifying information collected. Their responses were guaranteed to be used only for academic research and would not affect their career development, minimizing social desirability bias (Section 3.2, Page 16), “*All participants were given detailed information about the academic purpose of this study, and were assured their response would be confidential. Also, they were guaranteed that their individual response would not be identified in the results, and would not have any influence on their future career*”.

**Response quality control**: Invalid questionnaires were systematically excluded based on pre-determined criteria: (1) ≥3 missing items in any single scale; (2) identical responses to all items (indicating inattentive responding); (3) obvious logical contradictions (e.g., “0 years of service” but “1000+ flight hours”) (Section 3.2, Page 16).

(2) Statistical bias minimization

**Common Method Bias (CMB) control**: A two-pronged approach was adopted:

Post-hoc Harman’s single-factor test extracted 32.7% of total variance (below the 40% threshold), indicating no severe CMB (Section 4.1, Page 21), “*we first conducted a post-hoc Harman’s single-factor test using Amos 24.0. This test—retained for transparency as a widely used initial screening tool [122]—extracted 32.7% of total variance from the unrotated factor solution, well below the 40% threshold indicating severe CMB [149]*”.

Multi-factor confirmatory factor analysis (CFA) showed the four-factor model (SL, MW, WE, SP) fit excellently (χ²/df = 1.87, CFI = 0.97), while the single-factor model fit poorly (CFI = 0.63), further confirming no significant CMB (Section 4.1, Page 21), “*we performed multi-factor CFA in Amos 24.0. The theoretical four-factor model (spiritual leadership [SL], meaningful work [MW], work engagement [WE], service performance [SP]) exhibited excellent data fit: χ²/df = 1.87, goodness-of-fit index (GFI) = 0.92, normed fit index (NFI) = 0.95, comparative fit index (CFI) = 0.97, root mean square error of approximation (RMSEA) = 0.052, and root mean square residual (RMR) = 0.031. By contrast, the single-factor model (all 40 items loading onto one factor) showed poor fit: χ²/df = 8.49, GFI = 0.36, NFI = 0.61, CFI = 0.63, RMSEA = 0.155*”.

A marker variable (“frequency of using public transportation”) was employed, with an average correlation of r = 0.08 (*p* > 0.05) with core variables, verifying minimal shared variance attributable to CMB (Section 4.1, Page 25), “*We further employed a marker variable (a theoretically unrelated construct) to quantify and control for potential CMB, following Lindell & Whitney [150]. The marker variable—‘frequency of using public transportation’—was assessed on a 5-point Likert scale (1 = Never, 5 = Daily) and has no theoretical link to SL, MW, WE, or SP. The average correlation between the marker variable and core variables was r = 0.08 (p > 0.05), indicating minimal shared variance attributable to CMB*”.

**Missing data bias control**: We conducted Little’s Missing Completely at Random (MCAR) test, which yielded results (χ² = 38.72, df = 42, p = 0.601) confirming that missing data were completely random. (Section 3.2, Page 16), “*Little’s Missing Completely at Random (MCAR) test was further conducted to assess the missing data mechanism, with the results (χ² = 38.72, df = 42, p = 0.601) indicating that the missing data were completely random (MCAR). This statistical evidence justified the use of listwise deletion for handling invalid questionnaires [146]*”.

**Sensitivity analysis**: No significant differences in demographic (age, years of service) or core variables (SL, SP) were found between valid and excluded cases (t-values: 0.52–1.23, *p* > 0.22), confirming the robustness of the results (Section 4.1, Page 24), “*To confirm that missing data did not introduce systematic bias, we conducted a sensitivity analysis. Results revealed no significant differences between valid and excluded cases in key demographic variables (age: t = 1.23, p = 0.22; years of service: t = 0.98, p = 0.33) or core variables (spiritual leadership [SL]: t = 0.76, p = 0.45; service performance [SP]: t = 0.52, p = 0.60)*”.

7. **Report ethics approval and consent/confidentiality safeguards.**

**Response:** Thank you for your comment. We confirm the relevant information is fully reported in the revised manuscript as follows:

- The ethics approval (Page 14):

*“The materials, method and procedures performed in this study involving human participants conform to the 1964 Helsinki declaration and its later amendments or comparable ethical standards, and they were approved by the Human Research Ethics Committee of Shaanxi Normal University, and informed consent was obtained from all individual participants included in this study.”*

- Consent safeguards (Page 16-17, Section 3.2):

*“All participants were given detailed information about the academic purpose of this study, and were assured their response would be confidential. Also, they were guaranteed that their individual response would not be identified in the results, and would not have any influence on their future career”*

**Statistical reporting**

8. **Map each analysis to each RQ/hypothesis;**

**Response:** Thank you for your comment. We have explicitly mapped each hypothesis to its corresponding analytical method in the revised manuscript by adding targeted explanatory statements in the **statistical method section (Section 3.4, Statistics analysis)** and **mediation effect analysis section (Section 4.3, Mediation effects analysis** and **Bootstrap Results of Chain Mediating Effects)** to establish a clear **one-to-one** correspondence. The specific mapping details and revised manuscript citations are as follows:

(1) Revised manuscript supplementary statements for mapping

- Statistics analysis (Section 3.4, Page 19): “*Specifically, Pearson correlation and regression analyses were used to test the direct relationships in Hypotheses 1,2,3,5,6,8, while the bootstrap method in PROCESS Model 6 was adopted to verify the independent mediation effects (Hypotheses 4,7) and sequential mediation effect (Hypothesis 9).”*
- Mediation effects analysis (Section 4.3, Page 23): *As model 1 shows, spiritual leadership is positively related with meaningful work (b = 0.699, 95% CI = [0.642, 0.756], p < 0.001), supporting Hypothesis 2. In model 2, spiritual leadership is positively associated with flight attendants’ work engagement (b = 0.649, 95% CI = [0.457, 0.842], p < 0.001), which supports Hypothesis 6, and meaningful work positively affect flight attendant’s work engagement (b = 0.486, 95% CI = [0.263, 0.709], p <0.001), supporting Hypothesis 8. In model 3, spiritual leadership (b = 0.227, 95% CI = [0.087, 0.367], p < 0.01), meaningful work (b = 0.317, 95% CI = [0.161, 0.473], p < 0.001) and work engagement (b = 0.081, 95% CI = [0.005, 0.157], p < 0.05) all have positively influence on flight attendants’ service performance, which supports Hypothesis 1, Hypothesis 3 and Hypothesis 5 respectively. Up to now, Hypothesis 1, Hypothesis 2, Hypothesis 3, Hypothesis 5, Hypothesis 6 and Hypothesis 8 are proved to be true.”*.
- Bootstrap Results of Chain Mediating Effects (Section 4.3, Page 25)*:”* ***Path 1 (SL → MW → SP)****: The indirect effect was estimated as b = 0.222, 95% CI = [0.140, 0.316], p < 0.001, supporting* ***Hypothesis 4****.*

***Path 2 (SL → MW → WE → SP)****: The chain mediation effect was estimated as b = 0.028, 95% CI = [0.006, 0.062], p < 0.05, supporting* ***Hypothesis 9****.*

***Path 3 (SL → WE → SP)****: The indirect effect was estimated as b = 0.053, 95% CI = [0.012, 0.100], p < 0.01, supporting* ***Hypothesis 7****.”*

(2) Specific hypothesis-analysis method mapping with manuscript citations

- Pearson correlation analysis (Section 4.2) and regression analysis (Section 4.3, Table 2): *“Descriptive statistics analysis and correlations analysis for all variables are shown in Table 1, and the correlation coefficients of the main variables were statistically significant... above significant correlations confirm the relationships between the variables discussed in the hypothesis"* (Section 4.2);
- Mediation effects analysis (Section 4.3, Page 23): “*Based on the analytical strategies mapped to each hypothesis, we tested the direct and mediating effects after controlling for four types of covariates.”*

9. **Justify models;**

**Response:** Thank you for your comment. We have fully justified all statistical models and analytical methods in the revised **Section 3.4 Statistics analysis** with literature and methodological rationales as follows:

- Pearson correlation & regression analyses: Selected to test the direct relationships in Hypotheses 1,2,3,5,6,8, as the standard method for examining bivariate correlations and direct predictive effects among core variables (Section 3.4), “*Next, the descriptive statistics calculation and Pearson’s correlations were completed by SPSS (See Section 4.2 for the relevant results)…Specifically, Pearson correlation and regression analyses were used to test the direct relationships in Hypotheses 1,2,3,5,6,8, while the bootstrap method in PROCESS Model 6 was adopted to verify the independent mediation effects (Hypotheses 4,7) and sequential mediation effect (Hypothesis 9).*”.
- PROCESS Model 6: Specifically chosen for testing sequential chain mediation, as this model is designed for analyzing multiple mediators and serial mediation pathways, matching the research objective of verifying Hypothesis 9 (Section 3.4) , “*Last, PROCESS Model 6 was selected for testing the sequential chain mediation effect of meaningful work and work engagement, as this model is specifically designed for analyzing multiple mediators and serial mediation pathways [120]; Model 6 of the PROCESS with SPSS was employed to verify the mediating effect of meaningful work, and work engagement.*”.
- Bias-corrected percentile bootstrap method (5000 samples): Adopted for mediation effect assessment, as it is recommended for mediation testing due to robust performance with non-normal data distributions, and 5000 samples ensure the accuracy of confidence interval calculation (Section 3.4), “*The bias-corrected percentile bootstrap method [118, 119] was used in this study to assess the mediation effect, a method recommended for mediation testing due to its robustness to non-normal data distributions [119], which is that a significant mediating effect was indicated if bootstrap confidence interval (CI) does not include zero based on 5000 random samples [120, 121].*”.
- CFA (Amos 24.0): Employed to verify scale construct validity and exclude common-method bias, with model fit evaluated by the classic criteria recommended by Hu and Bentler [117] (Section 3.4), “*Then the confirmatory factor analysis (CFA) was excuted by Amos to varify the validity of each measure and to exclude the influence of common-method bias, referring to the fit indices of the model to the data recommended by Hu and Bentler [117]*”.

All model selections are tailored to the study’s research hypotheses and design, with rigorous methodological and literature support.

10. **Report assumptions/diagnostics;**

**Response:** Thank you for your comment. We have clearly reported all statistical assumptions and model diagnostics in the revised manuscript, as detailed below:

- Regression model fit assumptions/diagnostics (Section 3.4, Page 20): “*All regression analyses were pre-validated for compliance with basic statistical assumptions (residual normality and homoscedasticity) via SPSS diagnostic tools; no assumption violations were observed. Key model diagnostic indices (including R², F-value, and 95% CI) were reported for all analyses to verify model validity*”.

In addition, the bootstrap method (5000 samples) was used to test mediation effects based on 95% confidence intervals (Section 3.4, Page 19), and common method bias and missing data diagnostics were fully reported in Section 4.1 and Section 3.2, respectively.

- Mediation effect diagnostics (Section 3.4, Page 19): “*The bias-corrected percentile bootstrap method [118, 119] was used in this study to assess the mediation effect, a method recommended for mediation testing due to its robustness to non-normal data distributions [119], which is that a significant mediating effect was indicated if bootstrap confidence interval (CI) does not include zero based on 5000 random samples [120, 121].*”
- Common method diagnostics (Section 3.4, Page 19): “*Then the confirmatory factor analysis (CFA) was executed by Amos to verify the validity of each measure and to exclude the influence of common-method bias, referring to the fit indices of the model to the data recommended by Hu and Bentler [117]*”
- Missing data diagnostics (Section 3.4, Page 19): “*Little’s Missing Completely at Random (MCAR) test was further conducted to assess the missing data mechanism, with the results indicating that the missing data were completely random (MCAR).*”

11. **Provide effect sizes and confidence intervals (not only p-values);**

**Response:** Thank you for your comment. We have revised the manuscript to explicitly report effect sizes and 95% confidence intervals alongside statistical significance in the text, in addition to their presentation in the tables. Specifically, Cohen’s d was reported for independent-samples t-tests in the sensitivity analysis, and key results now include full information on R^2^(explained variance), path coefficients, and 95% bias-corrected confidence intervals for both direct and indirect effects, rather than only p-values, to improve the transparency and interpretability of the findings.

- The Sensitivity analysis (Section 4.1, Page 21): *“To confirm that missing data did not introduce systematic bias, we conducted a sensitivity analysis. Results revealed no significant differences between valid and excluded cases in key demographic variables (age: t = 1.23, p = 0.220, 95% CI = [-0.12, 0.50], Cohen’s d = 0.09; years of service: t = 0.98, p = 0.330, 95% CI = [-0.08, 0.46], Cohen’s d = 0.07) or core variables (spiritual leadership [SL]: t = 0.76, p = 0.450, 95% CI = [-0.10, 0.42], Cohen’s d = 0.05; service performance [SP]: t = 0.52, p = 0.600, 95% CI = [-0.15, 0.37], Cohen’s d = 0.04).”*
- Mediation effects analysis (Section 4.3, Page 23-24): “*As model 1 shows, spiritual leadership is positively related with meaningful work (b = 0.699, 95% CI = [0.642, 0.756], p < 0.001), supporting Hypothesis 2. In model 2, spiritual leadership is positively associated with flight attendants’ work engagement (b = 0.649, 95% CI = [0.457, 0.842], p < 0.001), which supports Hypothesis 6, and meaningful work positively affect flight attendant’s work engagement (b = 0.486, 95% CI = [0.263, 0.709], p <0.001), supporting Hypothesis 8. In model 3, spiritual leadership (b = 0.227, 95% CI = [0.087, 0.367], p < 0.01), meaningful work (b = 0.317, 95% CI = [0.161, 0.473], p < 0.001) and work engagement (b = 0.081, 95% CI = [0.005, 0.157], p < 0.05) all have positively influence on flight attendants’ service performance, which supports Hypothesis 1, Hypothesis 3 and Hypothesis 5 respectively. Up to now, Hypothesis 1, Hypothesis 2, Hypothesis 3, Hypothesis 5, Hypothesis 6 and Hypothesis 8 are proved to be true. It should be noted that, in model 3, the calculated effect of spiritual leadership on service performance is direct effect, while in model 4, the calculated effect of spiritual leadership on service performance is total effect (b = 0.529, 95% CI = [0.450, 0.609], p < 0.001).*”

12. **Address multiple comparisons if relevant;**

**Response:** Thank you for your comment. Our main analyses focused on testing a single structural chain mediation model with a fixed set of a priori hypotheses, and no extensive exploratory or post-hoc pairwise comparisons were conducted. Within this framework, correction for multiple comparisons (e.g., Bonferroni, FDR) was not required, as all tests were theory-driven and focused on the hypothesized paths.

For the sensitivity analysis, the independent-samples t-tests were conducted solely to verify random missingness by comparing valid and excluded cases on a small number of key demographic and core variables. These were confirmatory, descriptive checks rather than exploratory hypothesis tests. Thus, no adjustment for multiple comparisons was applied, as the purpose was to evaluate the robustness of missing-data handling rather than to conduct inferential tests of multiple research hypotheses.

13. **Explain missing-data handling and add sensitivity analyses where conclusions could change;**

**Response:** Thank you for your comment. We have clearly described the procedure for missing-data handling and supplemented the relevant sensitivity analyses to ensure the robustness of the findings, as detailed in the section on statistical bias minimization in our response to **Question 6**.

- **Missing data bias control**: We conducted Little’s Missing Completely at Random (MCAR) test, which yielded results (χ² = 38.72, df = 42, p = 0.601) confirming that missing data were completely random. (Section 3.2, Page 16), “*Little’s Missing Completely at Random (MCAR) test was further conducted to assess the missing data mechanism, with the results (χ² = 38.72, df = 42, p = 0.601) indicating that the missing data were completely random (MCAR). This statistical evidence justified the use of listwise deletion for handling invalid questionnaires [146]*”.
- **Sensitivity analysis**: No significant differences in demographic (age, years of service) or core variables (SL, SP) were found between valid and excluded cases (t-values: 0.52–1.23, *p* > 0.22), confirming the robustness of the results (Section 4.1, Page 24), “*To confirm that missing data did not introduce systematic bias, we conducted a sensitivity analysis. Results revealed no significant differences between valid and excluded cases in key demographic variables (age: t = 1.23, p = 0.22; years of service: t = 0.98, p = 0.33) or core variables (spiritual leadership [SL]: t = 0.76, p = 0.45; service performance [SP]: t = 0.52, p = 0.60)*”.

14. **Avoid causal/overgeneralized claims for observational designs.**

**Response:** Thank you for your comment. We have carefully revised the manuscript to eliminate all causal or overgeneralized claims consistent with the cross-sectional, observational study design, and have explicitly acknowledged the inherent limitations for causal inference in the revised content. Specifically, all descriptive language referring to the relationships between variables (e.g., spiritual leadership, meaningful work, work engagement, and service performance) has been adjusted from causal phrasing (e.g., “causes”, “leads to”, “affects”) to correlational and associative expressions (e.g., “is positively associated with”, “is related to”, “correlates with”) that align with observational research. Additionally, we have added a dedicated subsection (**5.4.1 Cross-Sectional Design: Limitations in Temporal Dynamics and Causal Inference**) to explicitly clarify that the study only verifies associative relationships among variables rather than definitive causal links, and further propose longitudinal research designs for future causal inference verification. All revisions ensure that the study’s conclusions are strictly constrained by its observational design with no overgeneralization.

**Minor points and suggested reference: Improve precision/consistency**

15. **Define abbreviations;**

**Response:** Thank you for your comment. We have defined all abbreviations in the List of Abbreviations section (Page 40).

16. **Standardize terminology;**

**Response:** Thank you for your comment. We have carefully reviewed and standardized all terminology throughout the manuscript to ensure consistency, accuracy, and alignment with common usage in the field. For instance, “coefficient α” has been revised to “Cronbach’s α” to ensure accuracy and conformity with standard statistical expression.

17. **Tighten long paragraphs.**

**Response:** Thank you for your comment. We have revised the manuscript to split overly long paragraphs, improve logical flow, and enhance readability while preserving all key content and arguments.
