# Supplementary material for: Spiritual leadership and service performance among Chinese flight attendants: The mediating effects of meaningful work and work engagement
Source: PLoS One. 2026 May 21;21(5):e0324405. doi: 10.1371/journal.pone.0324405 (PMC13193339; doi:10.1371/journal.pone.0324405)
Supplement: Attachment — Submitted filename: Response to reviewers_R4.docx [file pone.0324405.s006.docx]

Dear Professor Boshra A. Arnout and Dr. Abdallah,

Thank you for your insightful and constructive comments on our manuscript entitled “Spiritual leadership and service performance among Chinese flight attendants: The mediating effects of meaningful work and work engagement” (ID: PONE-D-25-18162). We greatly appreciate the editorial team’s guidance and positive feedback recognizing the value of our work, as well as Reviewer #3’s rigorous evaluation. **Reviewer #3 has confirmed that all comments from the previous round have been addressed, and has provided one minor suggestion regarding the framing of novelty and clarification of research contributions.** We have carefully undertaken comprehensive and targeted revisions in response to this point, with all amendments made in strict accordance with the publication standards and academic guidelines of PLOS ONE. All revised content is clearly marked with Track Changes in the revised manuscript, and supporting materials are supplemented as required. Below is our detailed point-by-point response to this comment, along with the corresponding revisions made to the manuscript.

**List of Responses**

**Responses Reviewer #3:**

**Reviewer #3:**

1. **While the authors clarified the theoretical framing (JCT + JD-R), the novelty remains somewhat overstated.**

**Response:** We appreciate this constructive comment and have revised the manuscript to tone down overstated claims of novelty. Specifically, in Page 6, Paragraph 1 (Introduction, the core statement of research novelty), we removed the absolute original expression “*this study is the first to investigate the aforementioned relationships simultaneously from the perspectives of organizational psychology, positive psychology, and occupational psychology*” and rephrased it to a more measured description: “*while prior studies have explored the individual links between spiritual leadership, meaningful work, work engagement, and service performance across various occupational contexts, this study* ***advances the literature*** *by contextual application, theoretical integration and methodological testing of these relationships among Chinese flight attendants from the combined perspectives of organizational psychology, positive psychology, and occupational psychology*”. We frame our research as an advancement of existing literature rather than an entirely original exploration.

2. **The claim of being “the first” to examine these relationships is too strong and should be softened.**

**Response:** We agree that the phrase “the first” is overly assertive and have fully revised all such absolute expressions in the manuscript with two key modifications:

- See Response to Comment 1 for more details (revision of “the first” to “advances the literature”).
- In Page 36, Paragraph 3 (Section 5.3 of the Discussion), we added restrictive qualifiers to the original statement “*this is* ***the first*** *study to confirm the “SL→MW→WE→SP” pathway in the civil aviation context*” and revised it to: “*this study* ***attempts to*** *empirically validate the sequential “SL→MW→WE→SP” mediation pathway in the civil aviation context with a large sample of Chinese flight attendants, filling the gap of applying dual-theory and chain mediation to flight attendants’ service performance research*” (revision of “the first” to “attempts to”).

3. **The contribution should be reframed more precisely as: Contextual (aviation/flight attendants), Integrative (combining JCT + JD-R), Methodological (chain mediation)**

**Response:** We have fully revised the research contribution section in strict accordance with your suggestion, and rephrased the original vague description: “*Corresponding contributions include: (1) integrating JCT-JD-R to validate the SL→MW→WE→SP model; (2) providing large-sample empirical evidence; (3) offering actionable strategies for airline management.*” into a precisely categorized three-dimensional statement aligned with the research context and design, as follows:

“*Corresponding contributions are categorized into three precise dimensions aligned with the research context and design, with clear differentiation between theoretical novelty and contextual application: (1)* ***Contextual contribution (application)****: Extending spiritual leadership and service performance research to the aviation industry, and providing targeted empirical evidence for Chinese flight attendants’ service performance improvement in the high-stress civil aviation context; (2)* ***Integrative theoretical contribution (novelty)****: Bridging JCT and JD-R to construct a dual-theory analytical framework, revealing how SL shapes flight attendants’ SP through psychological state and resource activation—this cross-theoretical integration constitutes the core theoretical novelty of the study; (3)* ***Methodological contribution (novelty)****: Employing chain mediation analysis to unpack the layered sequential psychological mechanism between SL and SP, and verifying the independent and joint mediating effects of MW and WE, which further validates the novel MW-WE sequential chain mediation mechanism in the leadership-performance relationship.*”

4. **Recommendation: Clarify what is theoretically new vs. what is contextually applied.**

**Response:** We have fully addressed this recommendation in the revised research contribution section (final paragraph of the Introduction, Page 7). As outlined in our response to Comment 3, we explicitly distinguished theoretical novelty from contextual application by appending the corresponding attributes in parentheses to each contribution dimension: the Contextual contribution is clearly labeled as (*application*, i.e., contextually applied), while the Integrative theoretical and Methodological contributions are both marked as (*novelty,* i.e., theoretically new). This explicit annotation directly clarifies the study’s theoretical novelty and contextual application in full alignment with your suggestion.
